# Progress and priorities for reproductive, maternal, newborn, and child health in Kenya: a Countdown to 2015 country case study

**DOI:** 10.1016/S2214-109X(17)30246-2

**Published:** 2017-07-14

**Authors:** Emily C Keats, Anthony Ngugi, William Macharia, Nadia Akseer, Emma Nelima Khaemba, Zaid Bhatti, Arjumand Rizvi, John Tole, Zulfiqar A Bhutta

**Affiliations:** a Centre for Global Child Health, Hospital for Sick Children, Toronto, ON, Canada; b Aga Khan University, Nairobi, Kenya; c Aga Khan University, Karachi, Pakistan; d Dalla Lana School of Public Health, University of Toronto, Toronto, ON, Canada

## Abstract

**Background:**

Progress in reproductive, maternal, newborn, and child health (RMNCH) in Kenya has been inconsistent over the past two decades, despite the global push to foster accountability, reduce child mortality, and improve maternal health in an equitable manner. Although several cross-sectional assessments have been done, a systematic analysis of RMNCH in Kenya was needed to better understand the push and pull factors that govern intervention coverage and influence mortality trends. As such, we aimed to determine coverage and impact of key RMNCH interventions between 1990 and 2015.

**Methods:**

We did a comprehensive, systematic assessment of RMNCH in Kenya from 1990 to 2015, using data from nationally representative Demographic Health Surveys done between 1989 and 2014. For comparison, we used modelled mortality estimates from the UN Inter-Agency Groups for Child and Maternal Mortality Estimation. We estimated time trends for key RMNCH indicators, as defined by Countdown to 2015, at both the national and the subnational level, and used linear regression methods to understand the determinants of change in intervention coverage during the past decade. Finally, we used the Lives Saved Tool (LiST) to model the effect of intervention scale-up by 2030.

**Findings:**

After an increase in mortality between 1990 and 2003, there was a reversal in all mortality trends from 2003 onwards, although progress was not substantial enough for Kenya to achieve Millennium Development Goal targets 4 or 5. Between 1990 and 2015, maternal mortality declined at half the rate of under-5 mortality, and changes in neonatal mortality were even slower. National-level trends in intervention coverage have improved, although some geographical inequities remain, especially for counties comprising the northeastern, eastern, and northern Rift Valley regions. Disaggregation of intervention coverage by wealth quintile also revealed wide inequities for several health-systems-based interventions, such as skilled birth assistance. Multivariable analyses of predictors of change in family planning, skilled birth assistance, and full vaccination suggested that maternal literacy and family size are important drivers of positive change in key interventions across the continuum of care. LiST analyses clearly showed the importance of quality of care around birth for maternal and newborn survival.

**Interpretation:**

Intensified and focused efforts are needed for Kenya to achieve the RMNCH targets for 2030. Kenya must build on its previous progress to further reduce mortality through the widespread implementation of key preventive and curative interventions, especially those pertaining to labour, delivery, and the first day of life. Deliberate targeting of the poor, least educated, and rural women, through the scale-up of community-level interventions, is needed to improve equity and accelerate progress.

**Funding:**

US Fund for UNICEF, Bill & Melinda Gates Foundation.

## Introduction

Since gaining independence in 1963, Kenya has become one of the most thriving nations in east Africa. Its population has more than quadrupled, alongside rapid economic growth,^1^ with ambitions for the country to reach a middle-income classification by 2030. The innovation and technology sector is one of the most advanced in Africa, with escalations in mobile phone use and launch of the M-Pesa currency in 2007. Despite these successes, poverty levels remain high across the country, and health indicators in Kenya have not kept pace with other sectors. December, 2015, marked the endpoint of the Millennium Development Goals (MDGs) and, despite progress in some areas, Kenya did not achieve MDG targets of reducing maternal deaths by three-quarters (to 147 per 100 000 livebirths) and reducing under-5 deaths by two-thirds (to 33 per 1000 livebirths). Increased rates of maternal and child mortality were noted in the early-MDG period from 1990 to 1999,^2^, ^3^ whereas neonatal mortality remained relatively unchanged. This mixed and largely slow progress was partly compounded by underinvestment in health care, along with inequalities that persisted across the country. Health care was also affected by political instability in 2007, and by the devolution of health functions, funds, and resources from the central government to county governments after 2010. Results from other reports show stark disparities in coverage of key reproductive, maternal, neonatal, and child health (RMNCH) interventions by region, wealth group, education level, and rural or urban classification.^4^, ^5^ To enable comparisons with neighbouring countries such as Tanzania and Malawi, which have surpassed their MDG 4 targets,^6^, ^7^ a robust and comprehensive analysis of the Kenyan RMNCH status was needed to inform decisions on policies and programming.

**Research in context**

**Evidence before this study**

For our systematic literature review, we used the following search terms, with text words (tw) and MeSH terms (mh), using the broad domains of population {reproduction[mh] OR reproduct*[tw] OR obstetric[tw] OR mothers[mh] OR maternal[tw] OR parental[tw] OR newborn[mh] OR neonate[tw] OR baby[tw] OR infant[tw] OR child[mh] OR toddler[tw] OR child*[tw] OR preschooler[tw] OR juvenile[tw] OR adolescent[mh] OR youth[tw] OR teen*[tw]}; intervention {policy[mh] OR procedure[tw] OR action[tw] OR program*[tw] OR guideline[tw] OR protocol[tw] OR strategy[tw] OR plan[tw] scheme[tw] OR “education, medical”[mh] OR medical education[tw] OR “cost sharing”[mh] OR cost sharing[tw] OR “food, fortified”[mh] OR “dietary supplements”[mh] OR “breast feeding”[mh] OR “public-private sector partnerships”[mh] OR finance*[tw] OR “insurance, major medical”[mh] OR “health education”[mh] OR health education[tw] OR “vitamin a”[mh] OR vitamin a[tw]}; outcome {morbidity[mh] OR morbidity[tw] OR incidence[tw] OR prevalence[tw] OR mortality[mh] OR mortality[tw] OR contraception[mh] OR contraception[tw] OR fertility[mh] OR fertility[tw] OR “pregnancy in adolescence”[mh] OR adolescent pregnancy[tw] OR “delivery, obstetric”[mh] OR “family planning services”[mh] OR family planning[tw] OR malnutrition[mh] OR malnutrition[tw] OR underweight[tw] OR overweight[tw] OR stunting[tw] OR obesity[mh] OR obesity[tw] OR wasting[tw] OR vaccination[mh] OR immunization[mh] OR immunization[tw] OR “measles vaccine”[mh] OR measles vaccine[tw] “vitamin a”[mh] OR vitamin a[tw] OR health services accessibility[mh] OR “cost-benefit analysis”[mh] OR cost effectiveness[tw] OR “health services misuse”[mh] OR abuse of health services[tw] OR “medical informatics”[mh] OR health information technologies[tw] OR “electronic health records”[mh] OR electronic health record[tw] OR diarrhea epidemiology, children[tw]}; and setting {kenya[mh] OR kenya[tw]}. Text words and MeSH terms were used in the following combinations: (a) population and intervention; (b) intervention and outcome; (c) population, intervention, and outcome; (d) a or b or c; and (e) a or b or c and setting. Relevant stakeholders, including WHO, World Bank, and Kenyan Ministry of Health representatives, were interviewed to procure additional information on policy, health systems, and health financing.

**Added value of this study**

To our knowledge, this is the first comprehensive, systematic assessment of reproductive, maternal, newborn, and child health (RMNCH) indicators and outcomes throughout the Millennium Development Goal (MDG) era (1990–15) in Kenya. We examined mortality trends and causes of maternal, neonatal, and child deaths. Data from national demographic health surveys allowed a robust time-trend analysis of key indicators at both the national and subnational level, including disaggregation of intervention coverage by county and household wealth. Additionally, we used a hierarchical linear modelling approach to assess the determinants of change in coverage for three interventions that span the continuum of care, to elucidate the positive and negative predictive factors of health-care service use. Using the Lives Saved Tool, we could link coverage and mortality, both retrospectively and prospectively, to understand the RMNCH interventions that saved the most lives during the MDG period and those that would have the greatest effect if coverage was scaled up to 2030.

**Implications of all the available evidence**

This study provides a detailed account of mortality trends, intervention coverage, equity, and the determinants of maternal and child survival through multivariable and lives-saved analyses. Evidence from this study can be used to aid governments at both the national and county level in prioritising health needs. It will allow the Kenyan Government to fill gaps in health care in terms of policy and programming, and to target specific subgroups. Further research is needed to understand how to appropriately address the inequities that persist in Kenya.

We did a comprehensive national and subnational analysis of change in key Countdown coverage indicators and outcomes between 1990 and 2015 to assess Kenya's progress towards achieving MDGs 4 and 5. To frame our analyses, we used a Countdown-adapted impact model (appendix) that is commonly used in other research of maternal, newborn, and child health.^8^ This impact pathway incorporates a health-systems approach to understanding changes in maternal and child health through the various Countdown-specific domains, which include health systems and policies, health financing, coverage, and equity. In addition to these specified Countdown areas, we examined a range of contextual factors that had the potential to affect maternal and child health in Kenya. The conceptual framework reflects our broader objectives; in-depth findings on health systems and policies, health financing, and the determinants of mortality change will be reported separately.

This study aims to determine estimates and trends of maternal, neonatal, and under-5 mortality; assess coverage of key RMNCH interventions, disaggregated by standard equity variables; and examine the effect of specific interventions on mortality using the Lives Saved tool (LiST). This case study was supported by the Countdown to 2015 consortium and used standard Countdown study methods.^4^

## Methods

### Data sources and statistical analysis

#### Literature review

We did a systematic review of all available electronic data published, in English, between Jan 1, 1990, and May 31, 2015, as well as unpublished data. All data pertained to the situation analysis of RMNCH in Kenya from 1990–2015, including information about socioeconomic development, relevant policies, programme strategies and interventions, and official reports about progress towards MDGs.

#### Mortality

We estimated trends and the average annual rate of reduction (ARR) of maternal, stillbirth, neonatal (≤1 month), and under-5 mortality for the period 1989–90 to 2014–15. Data sources are listed in the appendix.^2^, ^3^, ^5^, ^9^, ^10^, ^11^, ^12^, ^13^, ^14^ For each mortality outcome, we retained both modelled and sampled estimates when available. We modelled all cause-of-death data with the LiST. Projections of rates to 2030 were based on two scenarios: current trends, estimated with the observed ARR, and the accelerated ARR required to achieve the Sustainable Development Goal targets for maternal (70 per 100 000 livebirths), newborn (12 per 1000 livebirths), and child (25 per 1000 livebirths) mortality.^15^

#### Coverage indicators

To measure health outcomes and trends in maternal, newborn, and child intervention coverage in Kenya between 1989 and 2014, we analysed raw data from the Kenya Demographic and Health Surveys (K-DHS). These surveys provide comprehensive estimates at both national and subnational levels. Average annual rates of change for the periods 1989–2003, 2003–14, and 1989–2014 were calculated for key interventions to better visualise trends in coverage. All coverage indicators were defined according to Countdown to 2015 guidelines.^16^ Because of small sample sizes at the country level, we used Bayesian spatial models to estimate key RMNCH indicators for these small geographical areas. We estimated Bayesian posterior prevalence rates for K-DHS surveys in 2003, 2008–09, and 2014, using covariates and standardised procedures that are detailed elsewhere.^17^ We used R (version 3.2.5) and WinBUGS (version 14) to calculate the estimated prevalence, and ArcGIS10 (version 10) to create high-resolution maps for visualisation of coverage of key indicators across counties.

#### Multivariable analysis

We explored key correlates of RMNCH intervention coverage in the period with the greatest growth

(2003–14). We did ecological multivariable regression using county as the unit of analysis. Analysed outcomes included coverage of family planning for women married or in union, skilled birth assistance, and full immunisation of children aged 12–23 months. We chose these outcomes on the basis of their unique positions in the continuum of care and different delivery strategies. We modelled outcomes as absolute differences in Bayesian prevalence estimates calculated for each of the 47 counties (difference between 2014 and 2003). Because of a scarcity of available covariate data at baseline (2003), we examined potential determinants using best-estimate fixed values for 2014. We adapted a hierarchical modelling approach, as suggested by Victora and colleagues,^18^ for similar datasets. We conceptualised three levels of potential determinants for each outcome (appendix). At the distal level (level 3) we examined a range of socioeconomic factors; the intermediary (level 2) variables included health service accessibility factors; and the proximal (level 1) variables were individual, household, and behavioural factors that could affect coverage levels. We explored univariate distributions of outcome and predictors using mean or median, frequency or percent, and histograms, as appropriate. We estimated crude associations between the covariate and the change outcome (and further adjusted for baseline values of the coverage outcome) via ordinary least-squares regression, and evaluated linear slope coefficients and partial *R*^2^ values. Covariates that were significant at liberal cutoff (p<0·20) in the crude analysis were incorporated into a series of model-building strategies at the respective conceptual level. We used a combination of highest adjusted *R*^2^ modelling and backward elimination model-building strategies to determine the final set of covariates at each level; variables were retained if p values were less than 0·15. In line with Victora's modelling strategy,^18^ factors that were significant in the distal model were retained in the intermediate model (irrespective of statistical significance), and factors that were significant in the combined set were retained in the proximal model. We iteratively evaluated assumptions of ordinary least-squares regression, including homoscedasticity and linearity, and found no important violations. We examined deviance and influence statistics, and used Akaike and Bayesian information criteria and *R*^2^ values to assess final model fit. We examined multicollinearity among predictors using variance inflation factors, whereby variance inflation factors greater than 3 indicated highly collinear variables. The type 1 error rate was 0·05 and we did statistical analyses with SAS (version 9.4).

#### Equity data and methods

The equity analysis was based on the stratified coverage of 11 interventions across the continuum of care according to household level wealth index. The appendix provides definitions of indicators. Household wealth was divided into five standard quintiles (Q1–5) and was developed on the basis of asset indices. We also calculated a composite coverage index (CCI)^19^ to present an overall picture of intervention coverage in Kenya. A five-dot plot was developed to visualise differences in intervention coverage with respect to wealth quintiles. All analyses were done in Stata (version 12). Analyses were weighted with DHS sample weights to restrict variability among sampling of different regions and ensure data were representative of the population.

#### Lives Saved Tool

We used the LiST to model the effect of scale-up of RMNCH interventions on maternal, neonatal, and child mortality. The appendix provides a complete list of interventions selected for modelling. We obtained the current level of coverage for each intervention from the latest available K-DHS (2014) estimates. When coverage data were unavailable for any intervention, estimates were made on the basis of known coverage of other interventions, as described in the LiST manual. For interventions not available in DHS, we used default data sources (appendix). For the prospective LiST model, 2016 was used as the baseline year and coverage was scaled up in two pragmatic scenarios: from base year to 2025, and from 2025 to 2030. The first scenario assumed scale-up of all interventions at first pragmatic level of coverage until 2025. We defined realistic targets for each intervention: target coverage of 50% if current level was less than 30%, target coverage of 70% if current coverage was above 30% but lower than 70%, and target coverage of 90% if current coverage was above 70%. In the second scenario, we scaled up coverage of interventions from the level attained in the year 2025 to the next pragmatic level by 2030. We also did retrospective LiST analyses to understand the effect of specific interventions on the observed mortality trends between 1993 and 2014. We further assessed the effect of scale-up of 13 community-delivered and primary care interventions to 90% of the current coverage by 2030 in different wealth quintiles, on the basis of the K-DHS 2014 data (appendix). We used LiST to model the cause-of-death structure for maternal, neonatal, and under-5 deaths using coverage levels from DHS and default data sources, when applicable. We also calculated cause-of-death estimates by quintile of socioeconomic status by assuming changes in coverage from the national level to the levels measured in each of the wealth subgroups.^20^

#### Ethics

The institutions that commissioned, funded, or administered the surveys were responsible for all ethics procedures.

### Role of the funding source

The funders of the study had no role in study design, data collection, data analysis, data interpretation, or writing of the report. The corresponding author had full access to all the data in the study and had final responsibility for the decision to submit for publication.

## Results

**Figure 1:**
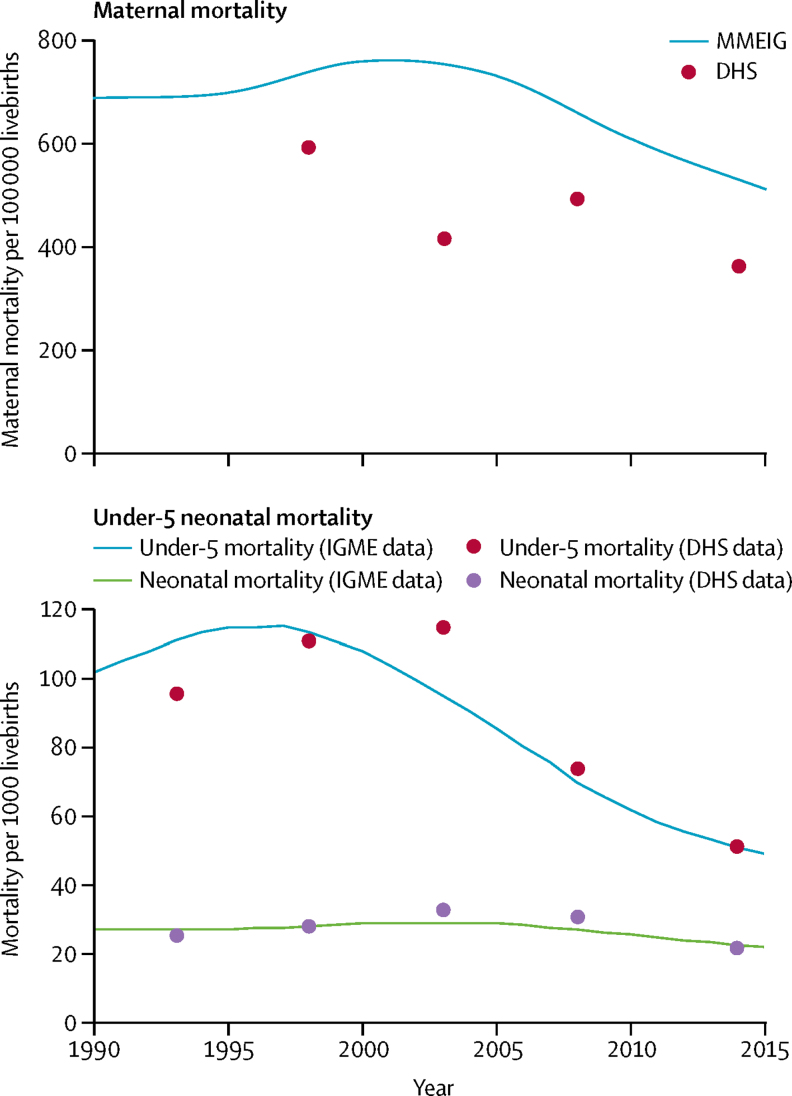
Trends in mortality rates, 1990–2015 Trends in maternal mortality per 100 000 livebirths. (B) Trends in mortality rates in under-5s and neonates. MMEIG=Maternal Mortality Estimation Inter-agency Group. DHS=Demographic and Health Surveys. IGME=Inter-agency Group for Child Mortality Estimation.

On the basis of data from the UN Maternal Mortality Estimation Inter-agency Group (MMEIG), maternal mortality declined from 687 deaths per 100 000 livebirths in 1990, to 510 per 100 000 in 2015, reflecting an overall average ARR of 1·2% and an absolute decline in mortality of 25·8% (figure 1). However, there was an increase in mortality from 1990 (687 deaths per 100 000 livebirths) to 2003 (768 deaths per 100 000 livebirths; ARR −0·9%) followed by a sharp decline thereafter at 3·4% annually (figure 1). DHS data for maternal mortality were available from 1998, and showed mixed progress between 1998 and 2015, with an overall decline in mortality of 38·6% (from 590 per 100 000 livebirths in 1998 to 362 per 100 000 in 2014; ARR 3·1%; figure 1).

Data from DHS for stillbirths (the number of pregnancy losses occurring after 28 weeks gestation) are scarce, but show that reductions in stillbirths have been minimal, declining from 1·4% of reported pregnancies in 2003, to 1·3% in 2014. Modelled data from *The Lancet* Stillbirth Group show a greater decline over the period for which data are available, from a rate of 26·4 per 1000 births in 2000, to 22·5 per 1000 in 2015. Despite a greater change, these data indicate that stillbirths still comprise 2·3% of all births in Kenya today.

Data from DHS surveys and the UN Inter-agency Group for Child Mortality Estimation (IGME) showed similar trends in under-5 and neonatal mortality (figure 1). On the basis of IGME, the overall average ARR was 2·9% for under-5 mortality and 0·8% for neonatal mortality between 1990 and 2015 (figure 1). Similar to maternal mortality, under-5 mortality increased throughout the early 1990s, with an ARR of −0·53%. Child deaths then substantially declined at an annual rate of 5·3% between 2000 and 2015. In absolute terms, under-5 mortality reduced by 51·7%, from 102·3 per 1000 in 1990, to 49·4 per 1000 in 2015. Neonatal mortality also increased between 1990 and 2003 (ARR −0·5%), but thereafter declined at 1·8% annually. Overall, neonatal mortality declined by 19·0%, from 27·4 per 1000 livebirths in 1990 to 22·2 per 1000 in 2015. Although trends are similar, DHS data showed later peaks in mortality and similar endline estimates for both under-5 (52 per 1000 livebirths) and neonatal (22 per 1000 livebirths) mortality, when compared with the modelled estimates. Graphs of neonatal, child, and maternal mortality indicate a similar pattern of increase in the 1990s, and a decline starting in the early 2000s (appendix).

The leading direct causes of death among mothers in 2014 were maternal haemorrhage (22·9%) and hypertensive disorders (15·4%; appendix). Indirect causes (30·6%), including AIDS-related indirect maternal deaths, also contributed substantially to overall maternal mortality, and became increasingly important between 1993 and 2014 (appendix). However, the proportion directly attributable to HIV/AIDS was only 2·4% of maternal mortality in 2014, a decrease from 9·7% in 2005, indicating that pre-existing disorders other than HIV/AIDS are becoming increasingly important when exacerbated by pregnancy, and that HIV management has greatly improved. Additional data sources have shown that coverage of all individuals receiving antiretroviral therapy has improved from 32% in 2010, to 59% in 2015, and that coverage of pregnant women receiving antiretroviral therapy for prevention of mother-to-child transmission of HIV has improved from 59% to 74%. The main causes of death in neonates were birth asphyxia, prematurity, and neonatal sepsis, together accounting for more than 75% of observed mortality in 2014 (appendix). Beyond the neonatal period, pneumonia and diarrhoea were responsible for 38·3% of child deaths in 2014 and were important causes of death throughout 1990 to 2015 (appendix). Whereas deaths from diarrhoea slowly declined from 2003 to 2015, other causes of under-5 deaths, including those from non-communicable diseases, infections, congenital abnormalities, and perinatal and nutritional conditions, are increasing. The most striking change in cause-of-death structure was affected by HIV/AIDS, which contributed to a substantial proportion of all-cause mortality before 2003, but has declined tremendously in recent years (appendix). The proportion of all under-5 deaths due to AIDS was highest in 1997 and 1998 (20·9%), and has declined to 2·4% in 2014. In adolescent mothers, abortion was the most predominant cause of death in 2014 (appendix). Because of data limitations, we were not able to track trends in adolescent maternal deaths.

**Figure 2:**
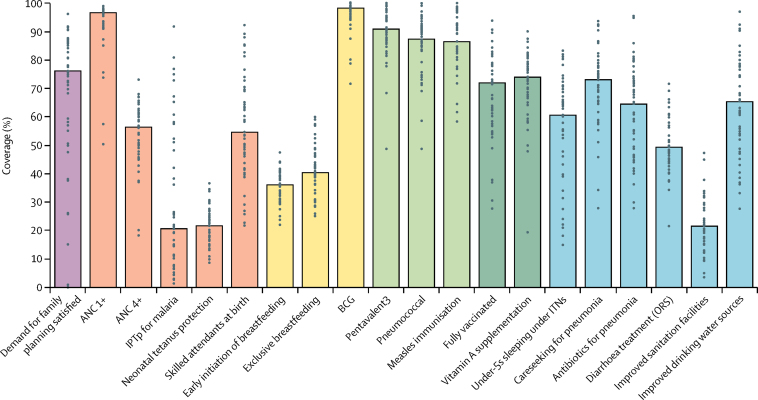
Intervention coverage across counties in 2014 Each dot represents coverage level per county (n=47), and bars indicate median coverage per intervention. ANC=antenatal care. ANC 1+=at least one antenatal care visit. ANC 4+=four or more antenatal care visits. IPTp=intermittent preventive treatment in pregnancy for malaria. ITN=insecticide-treated net. Pentavalent3=three doses of pentavalent vaccine. ORS=oral rehydration salts. DTP3=three doses of combined diphtheria, tetanus, and pertussis vaccine.

In 2014, coverage of essential RMNCH interventions across the continuum of care varied widely by intervention (figure 2). Median county-level coverage was highest for childhood immunisations and at least one antenatal care visit (ANC 1+), whereas low median coverage was noted for four or more antenatal care visits (ANC 4+), breastfeeding interventions, malaria and tetanus protection in pregnancy, and for improved sanitation facilities (figure 2). Although coverage across counties was fairly uniform for some interventions, such as those pertaining to breastfeeding and immunisations, other interventions showed wide regional disparities (figure 2). Unequal county-level coverage was typically associated with community-level or health-systems-based interventions, such as demand for family planning satisfied, skilled birth attendance, and use of insecticide-treated nets.

Tracing the coverage and penetration of key RMNCH interventions between 1989 and 2014 indicates an overall decline between the mid-1990s and 2003, followed by relative improvement for some interventions. This pattern is exemplified by several interventions along the continuum of care, as noted by the reversal of declining coverage (ie, the change from negative to positive average annual rate of change) that was seen between the earlier and later period (table 1). These key interventions represent differing delivery strategies, including facility-based, outreach-focused, or care-seeking, indicating influences on coverage that extend beyond health-system functioning.

**Table 1:** National-level trends in intervention coverage from 1989 to 2014

	**1989**	**1993**	**1998**	**2003**	**ARC 1989–2003**	**2008**	**2014**	**ARC 2003–2014**	**ARC 1989–2014**
Contraceptive prevalence (married or in union)	26·9%	32·7%	39·0%	39·3%	0·89%	45·5%	58·0%	1·70%	1·24%
Family planning needs satisfied	NA	48·0%	62·0%	61·6%	1·36%	64·0%	76·8%	1·38%	1·37%
ANC 1+	NA	93·5%	92·4%	88·0%	−0·55%	90·8%	95·7%	0·70%	0·10%
ANC 4+	NA	64·4%	60·5%	52·4%	−1·20%	47·2%	57·6%	0·47%	−0·32%
Neonatal tetanus protection	NA	48·4%	45·7%	50·1%	0·17%	51·0%	21·0%	−2·65%	−1·30%
Skilled birth assistance	50%	45·4%	44·3%	41·6%	−0·64%	43·8%	61·8%	1·84%	0·47%
Early breastfeeding	NA	60·1%	63·7%	56·8%	−0·33%	63·2%	34·1%	−2·06%	−1·24%
BCG	96·7%	96·3%	95·9%	87·3%	−0·67%	95·6%	96·7%	0·85%	0·00%
OPV3	92·4%	86·7%	80·8%	72·5%	−1·42%	87·5%	90·0%	1·59%	−0·10%
Measles vaccination	78%	83·8%	79·2%	72·5%	−0·39%	85%	87·1%	1·33%	0·36%
Full vaccination	72·8%	78·7%	65·4%	56·8%	−1·14%	77·4%	74·9%	1·65%	0·08%
Children (<5 years) using insecticide-treated nets	NA	NA	NA	4·6%	NA	46·7%	54·1%	4·50%	4·50%
Careseeking for pneumonia	NA	51·8%	57·3%	45·5%	−0·63%	55·9%	65·9%	1·85%	0·67%
Careseeking for diarrhoea	46·8%	40·9%	44·3%	29·7%	−1·22%	48·6%	57·6%	2·54%	0·43%
Diarrhoea treatment (oral rehydration salts)	21·1%	31·6%	36·9%	29·2%	0·58%	38·8%	53·8%	2·24%	1·31%
Improved sanitation facilities	NA	13·8%	16·2%	17·1%	0·33%	44·5%	24·7%	0·69%	0·52%
Improved water sources	NA	39·1%	30·6%	41·6%	0·25%	60·6%	66·9%	2·30%	1·32%

Data are for women of reproductive age (15–49 years) and children aged 6–59 months, including ARC (%) for the periods 1989–2003, 2003–14, and 1989–2014. ARC=average annual rate of change. NA=not applicable. ANC=antenatal care visit. ANC 1+=at least one antenatal care visit. ANC 4+=four or more antenatal care visits. OPV3=three doses of oral polio vaccine.

Our multivariable analyses identified several plausible drivers of improvements in intervention coverage over the past decade in Kenya. Table 2 summarises crude associations between the change outcomes (ie, family planning, skilled birth assistance, and full vaccination, from 2003 to 2014) and potential covariates, which are categorised into distal, intermediate, or proximal domains. Table 3 shows the final multivariable adjusted correlates of coverage change outcomes. Improvements in family planning were independently associated with less maternal and paternal illiteracy, greater equality for women, wider birth intervals, and more ANC 4+ (as a marker of health-care service use behaviour), after adjustment for family planning needs satisfied at baseline. The final multivariable model predicted 62% of the variation in change in family planning needs satisfied (p<0·0001), and included family planning needs satisfied at baseline, maternal illiteracy (β=−0·59; p<0·0001) and short birth interval (β=−0·72; p=0·0043). This finding indicates that the absolute change in family planning coverage between 2003 and 2014 declined by 0·6% points for every 1% increase in maternal illiteracy, and by 0·7% points for every 1% increase in shorter birth intervals. Analogous interpretations can be made for change in skilled birth assistance and full immunisation. In the crude analysis, a greater change in skilled birth assistance was linked to a range of determinants at a significance level of 0·05 (table 2). The final multivariable model predicted 74% of the variation in change in skilled birth assistance (p<0·0001) and retained baseline skilled birth assistance coverage, maternal education, gender equality for women, health worker density (doctors and nurses), national health insurance coverage, maternal age, and household size. Improvements in full vaccination were independently related to less maternal and paternal illiteracy, less poverty, wider birth intervals, fewer births, smaller households, and higher ANC 4+ coverage in crude analysis (p<0·05). The multivariable model included baseline full immunisation, maternal education, poverty levels, household size, birth intervals, and ANC 4+ (adjusted *R*^2^ 82%; p<0·0001).

**Table 2:** Crude associations between covariates with change in family planning, SBA, and full vaccination, from 2003 to 2014

	**Change in family planning(% point difference)**			**Change in SBA(% point difference)**			**Change in full vaccination(% point difference)**		
	β	p value	partial *R*^2^	β	p value	partial *R*^2^	β	p value	partial *R*^2^
**Distal: socioeconomic variables**									
Rurality	0·09	0·34	0·02	−0·11	0·25	0·02	−0·03	0·73	0·0008
Maternal illiteracy	−0·59	<0·0001	0·54	−0·39	<0·0001	0·26	−0·26	0·0016	0·06
Paternal illiteracy	−0·93	<0·0001	0·41	−0·80	<0·0001	0·30	−0·51	0·0007	0·07
Poverty	−0·04	0·72	0·003	−0·47	<0·0001	0·33	−0·24	0·0010	0·06
Female gender inequality	−0·07	0·0310	0·10	−0·12	<0·0001	0·26	−0·04	0·11*	0·02
Income inequality (0–1 rank)	0·45	0·10*	0·06	−0·05	0·86	0·0005	0·01	0·96	0·00002
**Intermediate: health-care resource variables**									
Health facility density (rate per 10 000 population)	−0·02	0·49	0·01	0·02	0·40	0·01	−0·006	0·83	0·0003
Health workforce density(rate per 10 000 population)	−0·001	0·43	0·01	−0·001	0·39	0·01	0·0006	0·70	0·001
Physician density (rate per 10 000 population)	0·18	0·37	0·02	0·31	0·0552*	0·06	0·21	0·24	0·01
Nurse density (rate per 10 000 population)	−0·004	0·44	0·01	−0·007	0·20*	0·03	0·001	0·79	0·0005
Midwife density (rate per 10 000 population)	..	..	..	0·01	0·83	0·0007	..	..	..
Availability of maternal units (% of total health facilities)	0·50	0·07*	0·07	0·56	0·0022	0·15	0·25	0·32	0·007
Availability of life-saving commodities (% of total health facilities)	..	..	..	0·51	0·07*	0·06	..	..	..
Availability of vaccines (% of total health facilities)	..	..	..	..	..	..	−0·12	0·65	0·002
Functional ambulances (rate per 100 000 population)	..	..	..	−0·03	0·0894*	0·05	..	..	..
Access to paved roads (% of total roads)	−0·14	0·60	0·007	0·21	0·33	0·02	−0·04	0·87	0·0002
Health spending per capita (US$)	−0·001	0·67	0·004	0·0008	0·72	0·002	−0·00006	0·98	0·000005
Coverage of National Health Insurance Fund (% population)	0·42	0·18	0·04	0·65	0·0026	0·14	0·40	0·10*	0·02
**Proximal: household, individual, and behavioural variables**									
Maternal age >35 years	−0·04	0·93	0·0002	0·84	0·0535*	0·06	0·50	0·19*	0·01
Birth interval <24 months	−0·95	0·0113	0·14	−0·73	0·0394	0·07	−0·92	0·0009	0·06
Parity (≥5 births)	..	..	..	−1·60	<0·0001	0·24	−0·75	0·0026	0·05
Household size (≥3 living children)	−0·04	0·89	0·0004	−1·04	<0·0001	0·22	−0·43	0·0098	0·04
Family planning needs satisfied†	..	..	..	0·40	<0·0001	0·21	..	..	..
ANC 4+†	0·40	0·0371	0·09	0·53	0·004	0·13	0·49	0·0014	0·06

Covariate data are %, unless otherwise stated. Crude estimates are adjusted for baseline (2003) levels of coverage of family planning, skilled birth attendance, and full vaccination, respectively. Female gender inequality measured via male:female ratio for completion of secondary school education. Income inequality measured using the GINI coefficient, where higher values indicate greater wealth inequality. Model coefficients can be interpreted per 1 unit change in the covariate with change in the outcome (eg, 1% increase in maternal illiteracy is associated with 0·6% point decrease in the change in family planning between 2003 and 2014. Or, for every additional health facility per 10 000 population, the absolute change in SBA coverage between 2003 and 2014 increases by 0.2% points. ANC=antenatal care. ANC4+=four or more antenatal care visits. SBA=skilled birth assistance.

* Statistically significant for model building (liberal p<0·20).

† Proxies for health-care seeking and use.

**Table 3:** Multivariable analysis of predictors of change in coverage for family planning, SBA, and full vaccination from 2003 to 2014

	**Adjusted coefficient (SE)**	**p value**
**Change in family planning (adjusted***R*^2^**=0·6203, p<0·0001)**		
Family planning needs satisfied (baseline)	−0·5737 (0·0743)	<0·0001
Maternal illiteracy (%)	−0·5862 (0·08)	<0·0001
Birth interval (<24 months, %)	−0·7179 (0·2381)	0·0043
**Change in SBA (adjusted***R*^2^**=0·7388, p<0·0001)**		
SBA (baseline)	−0·7009 (0·0975)	<0·0001
Maternal illiteracy (%)	−0·2342 (0·1016)	0·0261
Female gender inequality (ratio male:female)	−0·0689 (0·0309)	0·0311
Physician density (rate per 10 000 population)	0·2984 (0·1469)	0·0495
Nurse density (rate per 10 000 population)	−0·0082 (0·0041)	0·0553
NHIF coverage (% population)	0·6126 (0·2166)	0·0075
Maternal age (>35 years, %)	0·7565 (0·3148)	0·0217
Household size (≥3 living children, %)	−0·6482 (0·2224)	0·0062
**Change in full immunisation (adjusted***R*^2^**=0·8106, p<0·0001)**		
Full immunisation (baseline)	−0·8126 (0·0623)	<0·0001
Maternal illiteracy (%)	−0·1472 (0·0958)	0·1319
Poverty (%)	−0·1563 (0·0859)	0·0759
ANC 4+ (%)	0·3420 (0·1643)	0·0440
Household size (≥3 living children, %)	0·4060 (0·258)	0·1236
Birth interval (<24 months, %)	−0·7820 (0·2972)	0·0121

Change in coverage measured as percentage point difference. Female sex inequality measured via male:female ratio for completion of secondary school education. Model coefficients can be interpreted per 1 unit change in the covariate with change in the outcome—eg, 1% increase in NHIF coverage of the population is associated with 0·6% point increase in the change in SBA between 2003 and 2014, after adjusting for covariates and baseline SBA. Or, for every additional physician per 10 000 population, the absolute change in SBA coverage between 2003 and 2014 increases by 0·3 percentage points, after adjusting for covariates and baseline SBA. There was no statistically significant collinearity (variance inflation factors <3) between covariates. Variables were retained if p<0·15. SBA=skilled birth assistance. NHIF=National Health Insurance Fund. ANC4+=four or more antenatal care visits.

**Figure 3:**
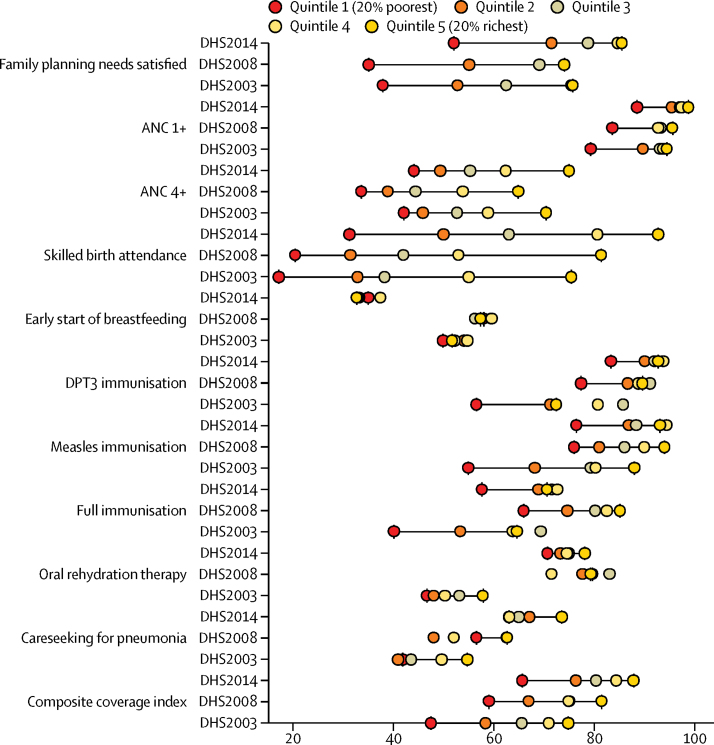
Intervention coverage and composite coverage index by wealth quintile for 2003, 2008, and 2014 DHS=Demographic and Health Surveys. ANC=antenatal care. ANC 4+=four or more antenatal care visits. DTP3=three doses of combined diphtheria, tetanus, and pertussis vaccine.

Disaggregation of intervention coverage by wealth quintile consistently shows greater coverage with higher household wealth (figure 3). Although coverage per quintile tended to improve over time, wide inequities persisted in 2014. In 2003, the absolute difference in coverage of skilled birth assistance was 58·4%, equating to coverage that was 4·4 times higher in the richest (Q5) households than the poorest (Q1), and in 2014 this absolute difference increased to 61·6%. Other disparities in coverage were noted for family planning needs satisfied and ANC 4+, two interventions for which the inequity gap remained unchanged from 2003 to 2014 (figure 3). Even when overall intervention coverage is high, such as for ANC 1+, patterns show bottom inequality (figure 3),^21^ indicating that the poorest women are disadvantaged compared with the rest of the population.

In addition to disparity by wealth quintile, mapping of intervention coverage at the county level revealed non-uniform improvements across Kenya, with substantial regional inequalities in coverage of family planning, skilled birth assistance, and full immunisation (appendix). For each intervention, counties comprising the northeastern, eastern, and northern Rift Valley regions had worsening, or consistently low, coverage over time relative to other areas of the country that showed steady improvements from 2003 to 2014. Change in county-level coverage of CCI between 2003 and 2014 further highlights the geographical disparity that continues to exist across regions of Kenya (appendix). Although some regions improved faster than others, improvement was ubiquitous from 2003 to 2008. However, several counties comprising the northeastern and Rift Valley regions had a decline in CCI coverage between 2008 and 2014.

LiST models estimated that 10 500 neonatal deaths, 42 000 under-5 deaths, and 1400 maternal deaths were averted through deployment of various interventions between 2003 and 2014. Management of labour and delivery and treatment of eclampsia accounted for 86% of the maternal lives saved (appendix). For neonates, optimal care and management during birth and the postnatal period accounted for 70% of all deaths averted, while preventive interventions, including those to prevent mother-to-child transmission of HIV, vaccinations, handwashing with soap, use of insecticide-treated nets, and improved sources of water, had the highest effect on reducing under-5 mortality (appendix). Curative interventions, such as antibiotic treatment of pneumonia and oral rehydration salts for treatment of diarrhoea, also contributed to a large proportion of deaths averted in children younger than 5 years.

**Figure 4:**
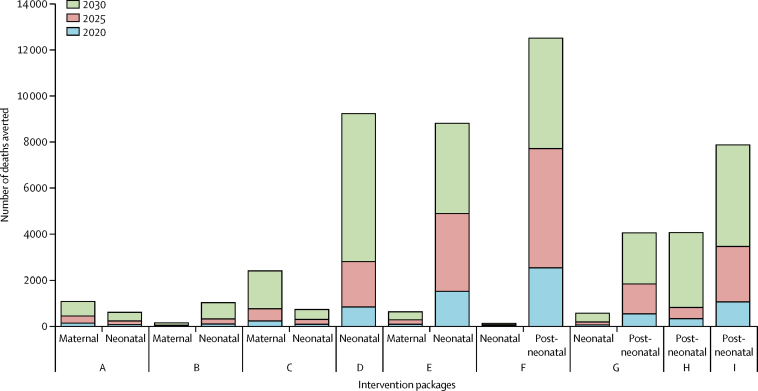
Projections of the number of maternal, neonatal, and post-neonatal deaths averted by specific intervention packages in 2020, 2025, and 2030 A=periconceptual and post-abortion care. B=expanded antenatal care package. C=optimal maternal nutrition during pregnancy. D=childbirth and immediate newborn care. E=postnatal care, including community newborn care. F=Integrated Management of Childhood Illnesses. G=Infant and Young Child Nutrition Package. H=Expanded Immunisation Package. I=water, sanitation, and hygiene interventions.

Prospective LiST models showed that by 2030, 83·5% of maternal deaths could be averted by pragmatic scale-up of a range of intervention packages, including periconceptual or post-abortion care and maternal nutrition interventions, while more than 6000 newborn lives could be saved with childbirth and newborn care intervention packages alone (figure 4). For children younger than 5 years, integrated management of childhood illness (defined as oral rehydration salts, zinc treatment for diarrhoea, antibiotics for dysentery, case management of pneumonia, insecticide-treated materials or indoor residual spray, therapeutic feedings for severe wasting, and treatment of moderate acute malnutrition) and water and sanitation (WASH) interventions would save the most lives (figure 4). LiST modelling by wealth quintile suggested that scale-up of community and primary care interventions to 90% coverage by 2030 would have the greatest effect among the poor, accounting for 70% of all neonatal and under-5 deaths averted in the two poorest quintiles (appendix).

## Discussion

Overall progress in improving maternal and child health in Kenya has been substantial, though variable throughout the MDG period. From the mid-1990s to the early 2000s, there was an increase in mortality, with maternal mortality, for example, reaching a high of 768 per 100 000 livebirths in 2003. This was mirrored by trends in neonatal mortality. Under-5 deaths peaked earlier, reaching a high of 115·4 per 1000 livebirths in 1996, but subsequently declined faster than maternal and neonatal deaths. ARR for maternal mortality in Kenya was roughly a third of the 3·4% annual reduction achieved in its southern neighbour, Tanzania. Additionally, ARR for under-5 mortality was nearly half of that achieved in Tanzania (5·0%)^6^ and Malawi (5·8%).^7^

Decline in mortality was slow because of insufficient coverage levels of key interventions throughout the early MDG period, coupled with the severe HIV/AIDS crisis that itself had a devastating effect on maternal and child health in Kenya. The reasons behind poor coverage are likely to be multifaceted, and largely related to the financial and social conditions of the country at the time. Results from our in-depth analyses of health systems, policies, and financing have alluded to this notion (unpublished data). Economic stagnation, the introduction of user fees, and a dearth of funds available for health-facility operating costs in the 1980s led to poor accessibility of health services by the public.^22^, ^23^, ^24^, ^25^ Additionally, the absolute number of health facilities could not keep pace with the rate of population growth,^26^ and the health workforce was widely under-represented across Kenya, highlighting logistical and financial barriers to access. Things improved after 2003, following a change in regime. To align with the global MDG agenda, there was a proliferation of RMNCH policies and programmes in Kenya, and increased funding for health that led to improvements in coverage levels of key interventions and reduction in mortality rates. However, gains in child health were far greater than gains in the health of mothers and newborn babies, an observation that reflects increased external funding specifically for this population subset and, consequently, the propagation of child health-related policies. Out-of-pocket spending continues to account for a substantial proportion of health spending in Kenya—a government tactic for management of facility operating costs that is undoubtedly contributing to the remaining inequity in intervention coverage between wealth groups and regions.

The results of our multivariable analyses also provided some insight into the determinants of change in intervention coverage over the last decade. *R*^2^ values ranged from 62% to 82%, indicating robust and well fitting models for each of the interventions examined. Maternal education appears to be a key driver of improved practices and coverage of major interventions across the continuum of care. Other important distal factors include reductions in poverty and female gender inequality, which were associated with changes in full immunisation and skilled birth assistance, respectively. Health-systems-related factors (health financing and workforce) were important only for skilled birth assistance; we anticipated this outcome, considering the importance of these factors for assisted delivery when compared with other interventions, such as contraceptive use and immunisations, which are more outreach-based in Kenya. Nurse density and improved skilled birth assistance showed a negative relationship, indicating that coverage of skilled birth assistance seemed to improve, despite potential decreases in nurse density during the same period. This paradoxical association might have been explained by increases in other health-systems factors, such as health insurance coverage. Among the more proximal factors, family size and structure appear to be important, with improvements in full immunisation and skilled birth assistance linked to fewer children, and full immunisation and family planning linked to wider birth intervals. Given that a small number of children could be an indication of delayed first pregnancy or more years of schooling for mothers, its association with our change outcomes is not surprising. Improvements in skilled birth assistance were also associated with increased maternal age, which could further reflect improved schooling. Notably, ANC 4+ was not a determinant of skilled birth assistance. A multicountry study^33^ on the influence of antenatal care on skilled birth assistance in rural Africa found similar results and indicated that, in Kenya specifically, the positive effect of antenatal care visits on skilled birth assistance was mediated by the content of the visits.^27^ Further research is needed to establish how to promote skilled birth assistance effectively among Kenyan women during routine antenatal care visits. More generally, we have shown health-care service use to be positively associated with additional seeking of services, since facility births was associated with family planning and both ANC 4+ and skilled birth assistance were linked to full vaccination coverage.

We have presented two estimates of maternal mortality: modelled estimates from MMEIG, and those sourced from K-DHS surveys. Although the sisterhood sampling methodology used in DHS has some limitations,^28^ such as recall bias, MMEIG estimates for 2015 were produced before the release of K-DHS 2014 data, indicating that this information was not captured within the model.^14^ As such, the K-DHS 2014 estimate of 362 maternal deaths per 100 000 livebirths could be more accurate than the 2015 MMEIG estimate of 510 per 100 000. Although this is an improvement, progress was still short of MDG targets, and a substantial acceleration of the ARR (from 3·4% to 11·7%) would be needed to meet the Sustainable Development Goal target of 70 per 100 000 livebirths by 2030.

Stillbirths in Kenya, which reduced more slowly than both maternal and under-5 mortality, might be a reflection of inadequate global attention and tracking of this issue. Of note, the survey-based estimates from DHS are widely under-reported because of recall bias, misclassification errors between stillbirths and early neonatal deaths, and selective omission by mothers. As such, these estimates should be interpreted with caution. The 2016 report by *The Lancet* Stillbirth Group^13^ revealed that stillbirth rates are higher in sub-Saharan African countries (28·7 per 1000 births) than in all other world regions, with most of these stillbirths being the result of intrapartum death (51·1%). Ending these preventable stillbirths in Kenya will require better birth and death registry data, and improved quality care during labour and delivery.

In fact, our LiST analysis has indicated that strengthened management during labour, delivery, and newborn care would have the greatest effect on RMNCH. Both preventive and curative interventions, especially those pertaining to the first day of life, will be essential in accelerating reductions in mortality. These interventions include maternal nutritional supplementation during pregnancy, management of pre-eclampsia, active management of the third stage of labour, chlorhexidine cord care, thermal and kangaroo mother care, neonatal resuscitation, and antibiotics to treat severe neonatal infection. Results from our health systems and policies analysis, which examined related maternal and newborn policy tracer indicators, revealed that several of these policies were not adopted until after 2010. This finding could partly explain why declines in mortality were accelerated in the latter half of the MDG period. Other Countdown country case studies, including Afghanistan,^29^ Ethiopia,^30^ Peru,^31^ and Tanzania,^6^ have also supported the prioritisation of interventions pertaining to the first day of life. In each case, these low-cost, life-saving interventions have been shown to prevent thousands of deaths each year. Although each of these interventions exists within the primary health-care system in Kenya, our cause-of-death analysis has indicated that too many mothers and babies are still dying from these preventable causes. This finding highlights the extensive gap in availability and provision of quality care during and beyond childbirth. Indeed, national coverage of postnatal care for mothers reached only 53% in 2014. A study done in Kwale district^32^ reported that only 33% of women received the appropriate postnatal care during the first 7 days after childbirth. The same study pointed to understaffing, high staff turnover, and overall insufficiency of knowledge and skills among health-care workers as further inhibiting the provision of quality postnatal care in district hospitals.^32^ However, opportunities are readily available through improvements in education initiatives, bolstering of the health workforce, and strengthening of labour, delivery, and post-partum services at the community level. The gains in child health noted in the past decade (2003–15) are a strong indication that, with appropriately targeted, cost-effective interventions, progress in maternal and neonatal health can also be accelerated.

Despite improvements in median coverage levels over time, substantial gaps in coverage between the poorest and richest households remain for several key interventions across the continuum of care, particularly for those that are health-systems based. On the basis of our health systems and policies analysis, fully functioning health systems (encompassing, for example, proper infrastructure, sufficient health workforce, and a consistent supply of medicines and commodities) seem to be unevenly distributed across Kenya, with poor and marginalised communities experiencing greater challenges to access because of logistical (eg, geographical) and financial barriers. Although the devolution of health services aims to correct some of these stubborn issues, more appropriate targeting and outreach is needed in the interim. As has been shown in several other parts of eastern and southern Africa, deployment of a cadre of community health workers for integrated community case management and other outreach activities (eg, post-partum home visits, RMNCH education) is an important avenue for improving accessibility and delivery of services.^33^ In fact, our LiST modelling shows that the scale-up of community-delivered interventions would have the greatest effect on the poorest households in Kenya. The proportion of post-neonatal deaths prevented by scale-up of community-delivered interventions is 49% for the poorest households, compared with only 7% for the richest. Effective implementation of these interventions would not only avert morbidity and mortality, but would also increase equity in coverage of life-saving interventions.

Kenya has supported a community health system since 2006, although integrated community case management was not included in this approach until 2012. Currently, 59 810 community health workers support 29% of the population across all 47 counties, with plans for scale-up to 260 000 by 2017.^33^ Although this is a step in the right direction in terms of reaching those in remote and marginalised areas, many challenges remain. First, monetary incentives are absent or extremely low (US$25 per month), when they are provided by non-governmental organisations.^33^ Second, commodity supplies for community health workers are not always in stock, making provision of quality care and service-use retention problematic.^33^ Overall coverage of interventions delivered by community health workers is currently below its target reach, which is a reflection of impediments to supply and demand, including poor implementation status of the strategy, and possibly poor staff morale, low availability of commodities, or insufficient recipient awareness.^33^ However, when there is strong capacity building for these programmes, evidence for impact has been shown: evaluation of the East Africa Maternal Newborn and Child Health Project, which trained and deployed community health workers in Kilifi county to deliver RMNCH information during home visits,^34^ found that mothers visited by community health workers were 5·1 times more likely to access postnatal services post-partum and 2·7 times more likely to use insecticide-treated bednets, whereas those not visited by community health workers had 2·3 times the risk of their newborn baby dying at home.^34^ Kenya's community health strategy, in partnership with the Ministry of Health, must continue to be strengthened for sustainable and widespread impact across all counties.

Although there are obvious logistical barriers to achieving 100% coverage of interventions among more remote and rural populations, urban slums retain their own unique set of challenges. In 2014, Kenya's population rapidly expanded to 43 million, from 28·7 million at the turn of the millennium,^3^ and in 2015, Kenya's urban population reached 26%.^35^ As a consequence, informal settlements, also known as slums, have proliferated and expanded. In Nairobi, a city that typifies rapid urbanisation, an estimated 60–70% of the population reside in a slum.^36^ These settlements are characterised by overt poverty, overcrowding, improper infrastructure, poor access to clean water and sanitation facilities, and high rates of communicable diseases, including HIV/AIDS infections.^37^ As such, slums are calling into question the so-called urban advantage that was previously thought to exist between rural and urban counterparts. Results from other studies^37^, ^38^ have shown substantially lower immunisation coverage among residents of informal settlements of Nairobi, with especially poor coverage of measles and polio vaccines. Increased risks of malnutrition, diarrhoea, and other infections, combined with low uptake of preventative measures such as vaccinations, indicate that these children are at a distinct disadvantage and underscores the importance of reaching underserved pockets through unique and improved service delivery that will overcome typical barriers to use, such as distance and cost. As identified in the recent *Lancet* Series on slum health,^39^, ^40^ improved and targeted research on informal settlements is needed to ensure development of a proper evidence base for local policy. Kenya must account for the changing urban landscape through targeted policies and programmes that distinguish between the urban wealthy and poor.

In addition to contending with the health of urban slum dwellers, Kenya is home to Dadaab refugee camp. Situated in Garissa county, Dadaab is populated by more than 345 000 individuals, making it the largest refugee camp in the world and the equivalent to Kenya's third largest city.^41^ Although the camp is financed externally, inhabitants experience similar challenges to those living in urban slums.^42^ Dadaab is situated in an insecure area, creating the added challenge of seeking appropriate health services, especially for obstetric care.^43^ Moving forward, Kenya will need to ensure that high-quality services are extended to everyone, including its vast refugee population.

Our study has several limitations to note. Although we used robust methods within our multivariable analyses to understand the determinants of change in coverage over time, our analysis at the subnational level was limited by sample size. Devolution from the province to the county level took place in 2013, indicating that the 2014 K-DHS was powered for the county, but our 2003 estimates had to be extrapolated through Bayesian analysis. As such, observations per county were far fewer for the earlier survey, making them less reliable. Any unexplained variance was probably the result of unmeasured variables, including those more distal to the individual, such as the political context, economic and social security, and other such country-level factors. There is the issue of ecological fallacy that arises when aggregate-level estimates do not necessarily apply to the individual, a notion that should be considered with such analyses, in which the nuances of several distinct regions in Kenya might be overlooked with the use of grouped estimates.

Although the DHS datasets are a reliable source on which to base our analysis, sampling from several important regions, including all former northeastern province and other northeastern districts (Turkana, Samburu, Isiolo, Marsabit, and Moyale), did not begin until 2003. The northeastern province is largely nomadic, sparsely populated, and has been associated with regional instability due to sporadic conflict between resident tribes and clans and overall persistent insecurity.^44^ As such, a fairly substantial data gap exists for an area that has faced social, economic, and geographical challenges and, along with this dearth of information, has been left behind in terms of having accessible resources for health and wellbeing. As we have shown, more focused initiatives are required for the northeastern region in particular, so that it can attain the maternal and child health gains evident for the rest of the country.

The MDGs galvanised action for improving maternal and child health in Kenya, and much was done to attain the targets set out in the year 2000, with ongoing efforts today. Progress can be attributed to a multitude of factors, including strengthened commitment by the Kenyan Government, increased funding for health, proliferation of related policies and programmes, and a focus on the devolved state. A summary of key messages can be found in the panel. Although more work needs to be done to eliminate the inequality that remains for the rural, poorest, and least educated, the 2030 Sustainable Development Goals are more achievable now than ever.

Panel **Key messages**

• Insufficient coverage levels of high-impact interventions contributed to the plateauing of decline in maternal, newborn, and child mortality in Kenya. However, great efforts have been made to reverse the negative trends in coverage and mortality observed in the early Millennium Development Goal period.
• Declining maternal illiteracy is associated with improved coverage levels for interventions across the continuum of care.
• There are effective interventions pertaining to the first day of life that should be packaged for implementation in the health system. The primary focus should be on childbirth, newborn, and postnatal interventions to improve neonatal and maternal survival.
• Interventions to address the basic needs of children, including immunisations, infant and young child feeding practices, and management and treatment of diarrhoea and pneumonia, should be scaled up rapidly because they have potential for eliminating preventable child deaths.
• Improved and equitable progress will be achieved through implementation of community-level interventions to reach marginalised people. The Kenyan Government should invest in strengthening the existing Community Health Volunteer platform.
• Analyses in future studies should differentiate between the rural, urban wealthy, and urban poor populations in Kenya.
