# Supplementary material for: Progress and priorities for reproductive, maternal, newborn, and child health in Kenya: a Countdown to 2015 country case study
Source: Lancet Glob Health. 2017 Jul 14;5(8):e782–95. doi: 10.1016/S2214-109X(17)30246-2 (PMC5599303; doi:10.1016/S2214-109X(17)30246-2)

THE LANCET
Global Health

Supplementary appendix

This appendix formed part of the original submission and has been peer reviewed. We post it as supplied by the authors.

Supplement to: Keats EC, Ngugi A, Macharia W, et al. Progress and priorities for reproductive, maternal, newborn, and child health in Kenya: a Countdown to 2015 country case study. *Lancet Glob Health* 2017; **5:** e782–95.

Understanding Progress and Setting Priorities on Reproductive, Maternal, Newborn, Child, and Adolescent Health in Kenya: A Countdown to 2015 Country Case Study

Web Appendix

Table 1: Data sources.

**Analysis**	**Source**
RMNCAH coverage indicators	K-DHS 1989<br>K-DHS 1993<br>K-DHS 1998<br>K-DHS 2003<br>K-DHS 2008/09<br>K-DHS 2014
Neonatal mortality<br>Under-five mortality	IGME 1989-2014
	K-DHS 1988-2014
Maternal mortality	UN MMEIG 1990-2015
	K-DHS 1998-2014
Stillbirth	Lancet Stillbirth Group 2000-2015
	K-DHS 2003-2014
Cause of death	LiST modeling, 1993-2014
Equity	K-DHS, 2003-2014

IGME = UN Inter-Agency Group for Child Mortality Estimation; IHME = Institute for Health Metrics and Evaluation; MMEIG = WHO Maternal Mortality Estimation Inter-Agency Group; LiST = Lives Saved Tool; K-DHS = Kenya Demographic Health Survey

Table 2: Definition of indicators and CCI used in equity analysis.

Indicator		Definition
**FPS**	Family planning needs satisfied	Percentage of currently married fecund women who say that they do not want any more children or that they want to wait 2 or more years before having another child, and are using contraception (met need for contraception divided by the demand).
**ANCS**	Antenatal care visit by skilled provider	Proportion of mothers who were seen by a skilled health provider for at least one antenatal care visit during last pregnancy.
**ANC4**	4+ antenatal care visits	Proportion of mothers who had at least 4 antenatal care visits during last pregnancy.
**SBA**	Skilled birth attendant	Proportion of mothers who had their delivery assisted by a skilled health professional.
**EIBF**	Early initiation of breastfeeding	Proportion of newborns put to the breast in their first hour of life.
**ITNC**	Insecticide treated bed net for children	Proportion of children aged 0–59 months who slept under an ITN the night before the interview.
**DPT3**	DPT immunization	Proportion of children aged 12-23 months who received three doses of DPT vaccine.
**MSL**	Measles immunization	Proportion of children aged 12-23 months who received a dose of measles vaccine.
**FULL**	Fully immunized children	Proportion of children aged 12-23 months who received three doses of DPT and polio vaccines and one dose of BCG and measles vaccines.
**VITA**	Vitamin A supplementation	Proportion of children aged 6–59 months who received at least one high dose of vitamin A supplement in the previous six months.
**CPNM**	Care seeking for pneumonia	Proportion of children aged 0–59 months with suspected pneumonia taken to an appropriate health provider.
**ORT**	Oral rehydration therapy	Percentage of children aged 0–59 months with diarrhea in the previous two weeks who received oral rehydration therapy (packets of oral rehydration salts, recommended home solution, or increased fluids) and continued feeding.
**CCI**	Composite coverage index	CCI is calculated as the weighted average of coverage of a set of eight preventive and curative interventions; it gives equal weight to four stages in the continuum of care: family planning, maternal and newborn care, immunization, and case management of sick children. The following expression is used to obtain the estimate: where FPS is demand for family planning satisfied, SBA is skilled birth attendant, ANCS is antenatal care with skilled provider, DPT3 is three doses of DPT vaccine, MSL is measles vaccination, BCG is BCG vaccination, ORT is oral rehydration therapy and continued feeding for children with diarrhoea, and CPNM is care seeking for children with suspected pneumonia. $CCI = {}^{1}/_{4}\left(FPS + \frac{SBA+ANCS}{2} + \frac{2DPT3+MSL+BCG}{4} + \frac{ORT+CPNM}{2}\right)$

Table 3: Interventions utilized within LiST modelling, by package.

Packages	Interventions within Package
Periconceptual and post abortion care	Safe abortion services
	Post abortion case management
	Ectopic pregnancy case management
	Folic acid supplementation or fortification
Expanded antenatal care package	Syphilis detection and treatment
	Tetanus toxoid
	MgSO4 management of pre-eclampsia
	Diabetes screening and management
	Hypertensive disease case management
	Malaria case management
	Screening for fetal growth restriction and appropriate management
	IPTP: pregnant women protected via intermittent preventive treatment of malaria during pregnancy or by sleeping under an ITN
Optimal maternal nutrition during pregnancy	Calcium supplementation
	Protein energy supplementation
	Multiple micronutrient supplementation
Childbirth & immediate newborn care	Antibiotics for PRoM
	Labour and delivery management
	Clean birth practices at home
	Antenatal corticosteroids for preterm labor
	Immediate assessment and stimulation
	Neonatal resuscitation
	Active management of 3rd stage of labor
	MgSO4 management of eclampsia
	Induction of labour to prevent births at or beyond 41 completed weeks
Postnatal care (including community newborn care)	Preventive postnatal care (healthy practices and illness detection)
	Thermal care
	Kangaroo mother care
	Chlorhexidine
	Case management of severe neonatal infection
	Full supportive care for prematurity
	Maternal Sepsis case management
Integrated management of childhood illnesses (IMCI)	ORS
	Zinc for treatment of diarrhea

	Antibiotics for dysentery
	Case management of pneumonia
	Insecticide treated materials or indoor residual spraying
	Therapeutic feeding for severe wasting
	Treatment of moderate acute malnutrition
Infant and young child nutrition package	Exclusive breastfeeding till 6 month
	Complementary feeding education and supplementation
	Vitamin A supplementation
	Zinc supplementation
Expanded immunization package	Hib vaccine
	Measles vaccine
	DPT vaccination
	Rotavirus vaccine
	Pneumococcal vaccine
WASH interventions	Use of improved water source within 30 minutes
	Improved sanitation - Utilization of latrines or toilets
	Hand washing with soap
	Hygienic disposal of children's stools

Table 4: Default data sources used for LiST modeling, where coverage/outcome estimates could not be taken from DHS.

Section	**Indicator**	**Source of Default Data**
Health status, mortality, and economic status	Baseline child mortality (neonatal mortality rate and under-five mortality rate)	The UN Inter-Agency Group for Child Mortality Estimation (IGME)
Health status, mortality, and economic status	Percent of child deaths by causes	Liu L, Oza S, Hogan D, Perin J, Rudan I, Lawn JE, Cousens S, Mathers C, Black RE. 2014. "Global, regional, and national causes of child mortality in 2000–13, with projections to inform post-2015 priorities: an updated systematic analysis". Lancet. Epub Oct 1, 2014.
Health status, mortality, and economic status	Baseline maternal mortality	Trends in maternal mortality: 1990 to 2013. Estimates by WHO, UNICEF, UNFPA, The World Bank and the UN Population Division. Published 2014.
Health status, mortality, and economic status	Percent of maternal deaths by causes	Say L, Chou D, Gemmill A, Tuncalp O, Moller A, Daniels J, Gulmezoglu AM, Temmerman A, and Alkema L. 2014. "Global causes of maternal death: a WHO systematic analysis". Lancet Global Health. Epub May 6, 2014.
Coverage	WASH (improved water and improved sanitation)	WHO/UNICEF Joint Monitoring Program (JMP) for Water Supply and Sanitation
Coverage	Vaccines	WHO/UNICEF coverage estimates
Coverage	Vitamin A supplementation	UNICEF The State of the World's Children report
Coverage	Other coverage indicators	National surveys, including Demographic and Health Surveys (DHS)

Table 5: List of community-delivered interventions for LiST scale-up.

	**Intervention Type**
1	ANC
2	Breastfeeding promotion
3	Complementary feeding promotion
4	Vitamin A supplementation
5	Promotion of hand washing practices
6	Chlorhexidine cord care
7	Thermal care
8	ITN and IRS for children under 5 years
9	ORS
10	Zinc for diarrhea treatment
11	Oral antibiotics for treatment of NN infections
12	Oral antibiotics for treatment of pneumonia
13	SAM management

Figure 1: Kenya Countdown case study conceptual framework.

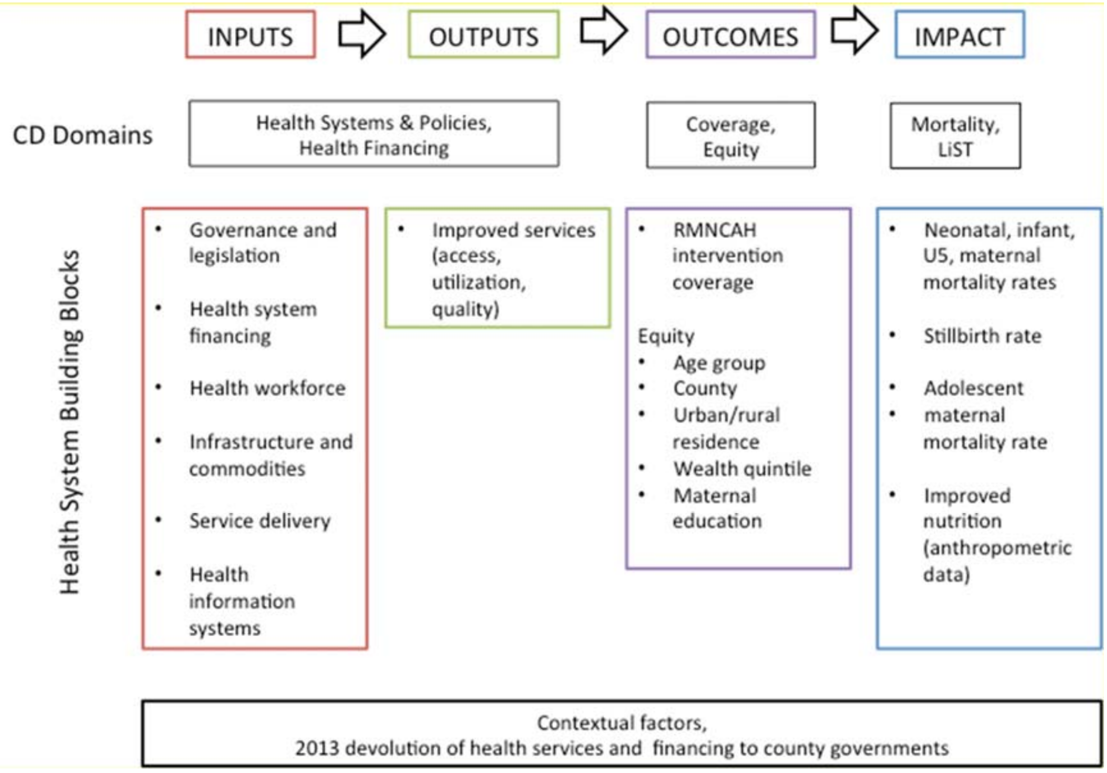

Figure 2a-c: Mapping of exploratory variables for multivariable analysis of change in family planning (a), skilled birth attendance (b), and full immunization (c).

a) Family planning conceptual framework.

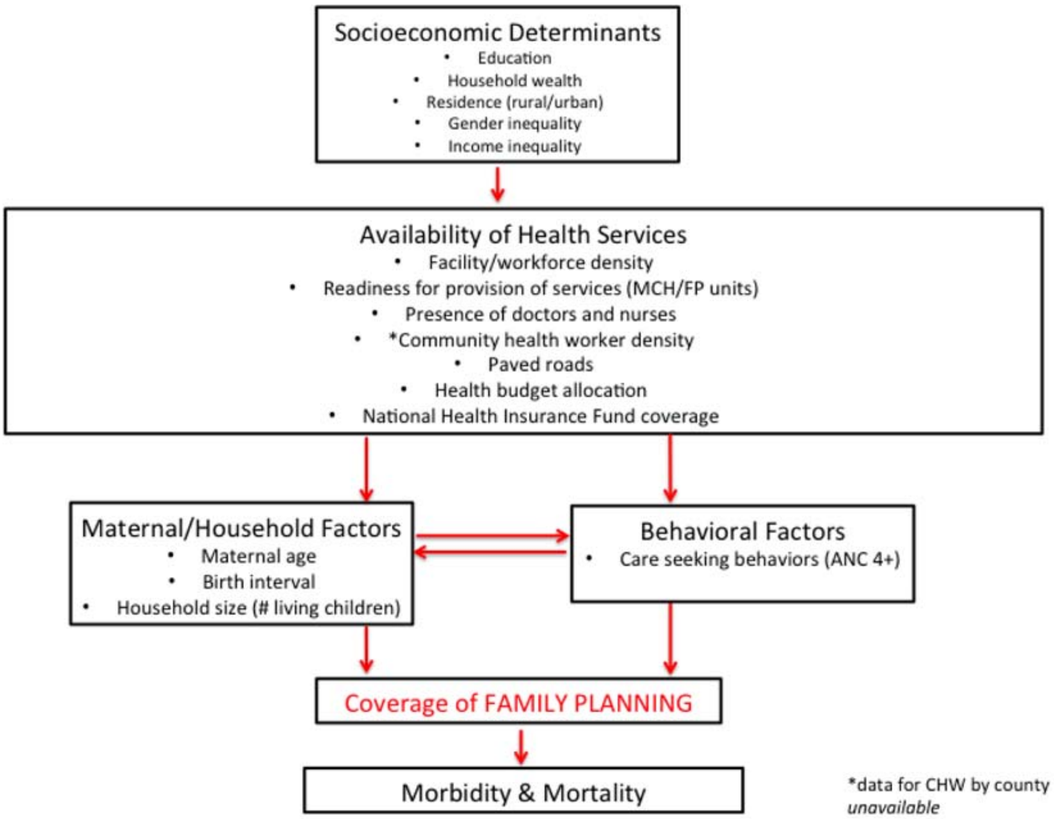

b) Skilled birth attendance conceptual framework.

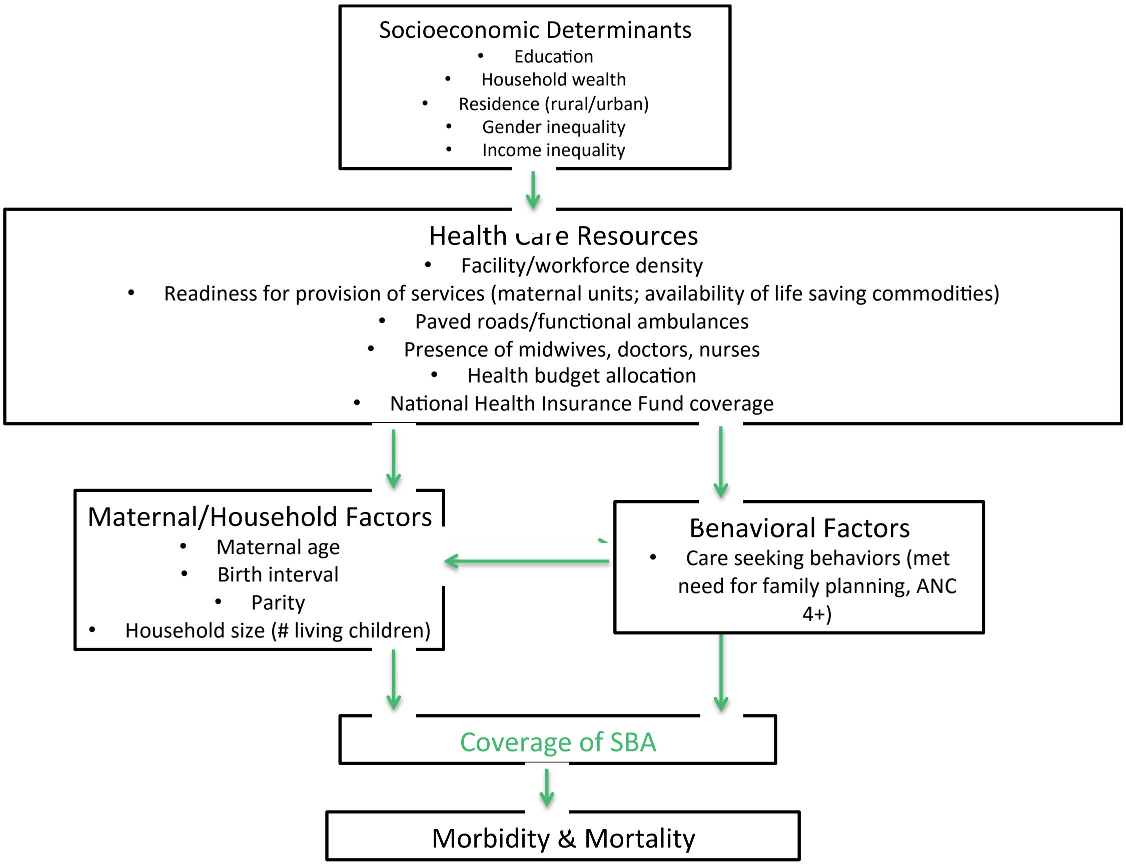

c) Full immunization conceptual framework.

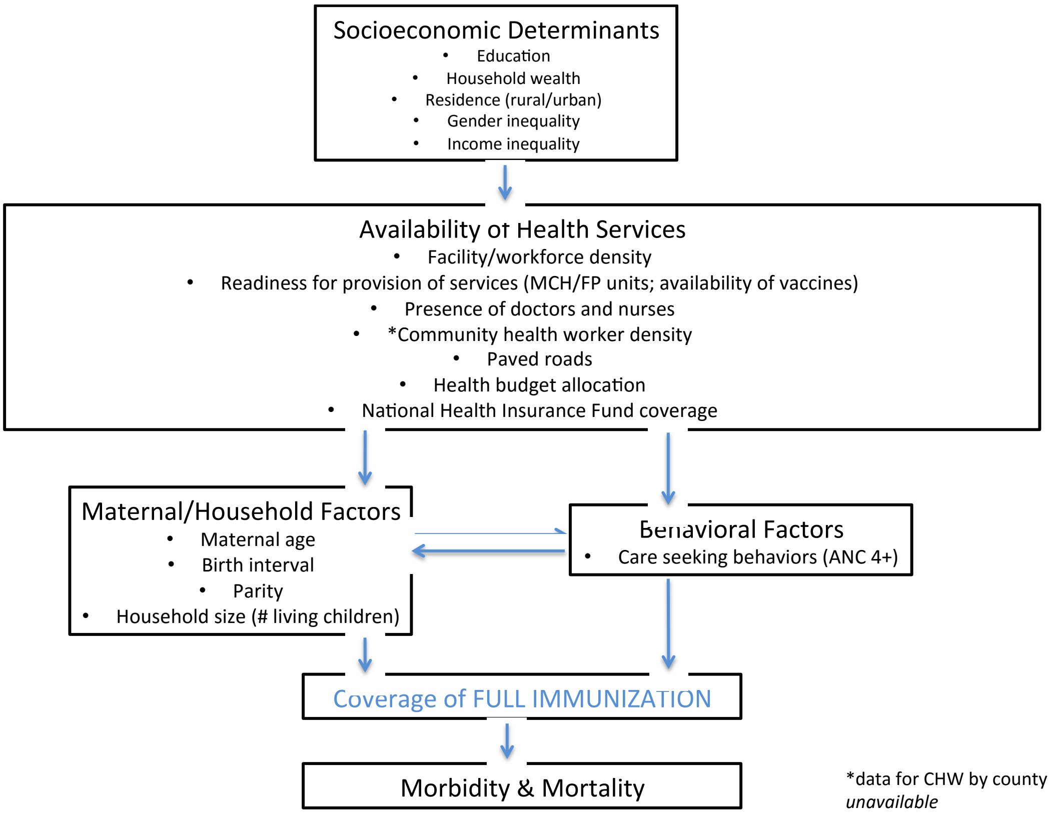

*data for CHW by county *unavailable*

Figure 3: Maternal, neonatal, and under-five mortality ratios in Kenya for the years 1990 to 2015.

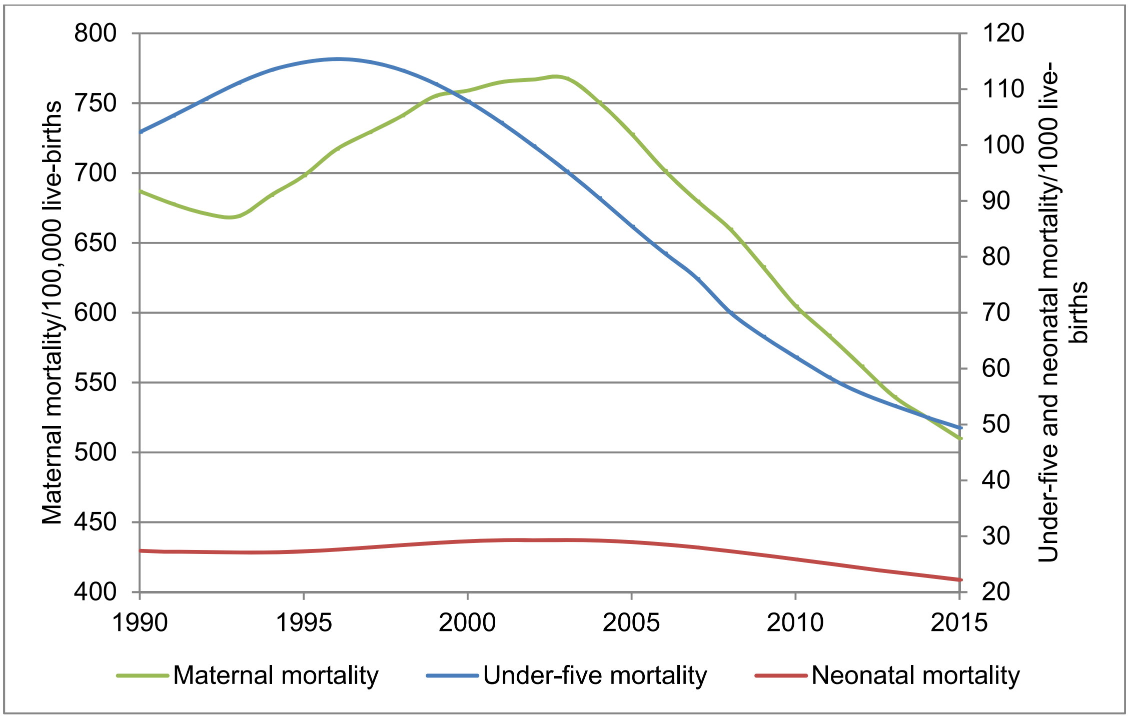

Figure 4: Causes of death among mothers, neonates, under-five (U5) children, and adolescent mothers in 2014.

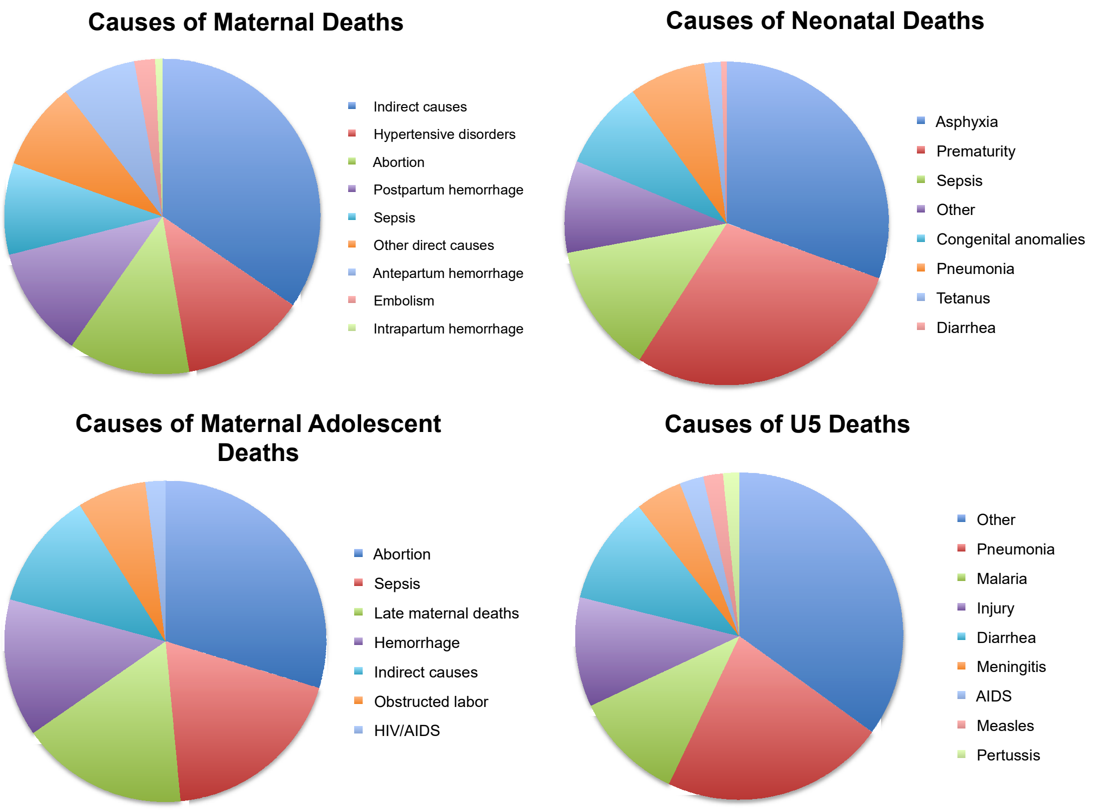

Figure 5: Trends in cause-specific maternal mortality for the period 1993 to 2014.

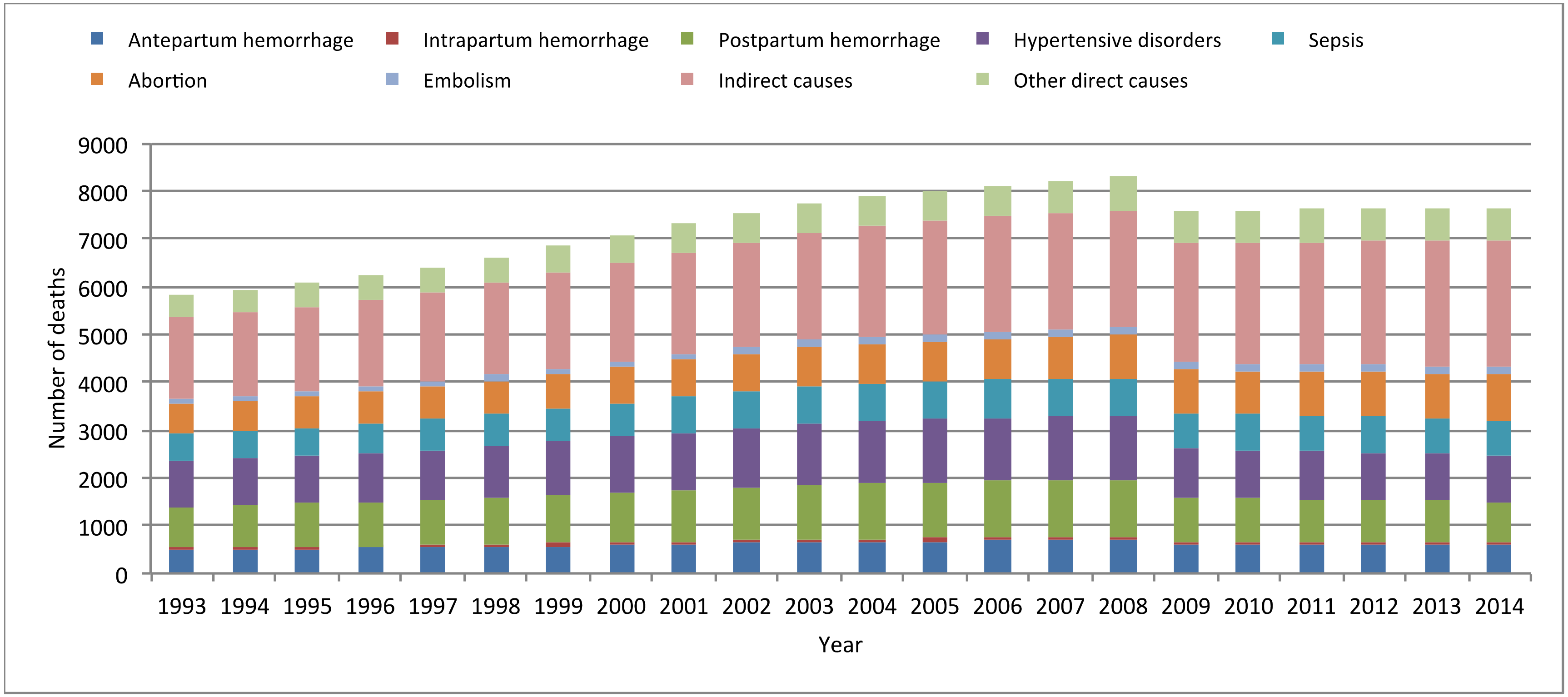

Indirect causes = medical disorders, HIV-related maternal deaths, and all other indirect causes.

Figure 6: Trends in cause-specific neonatal mortality for the period 1993 to 2014.

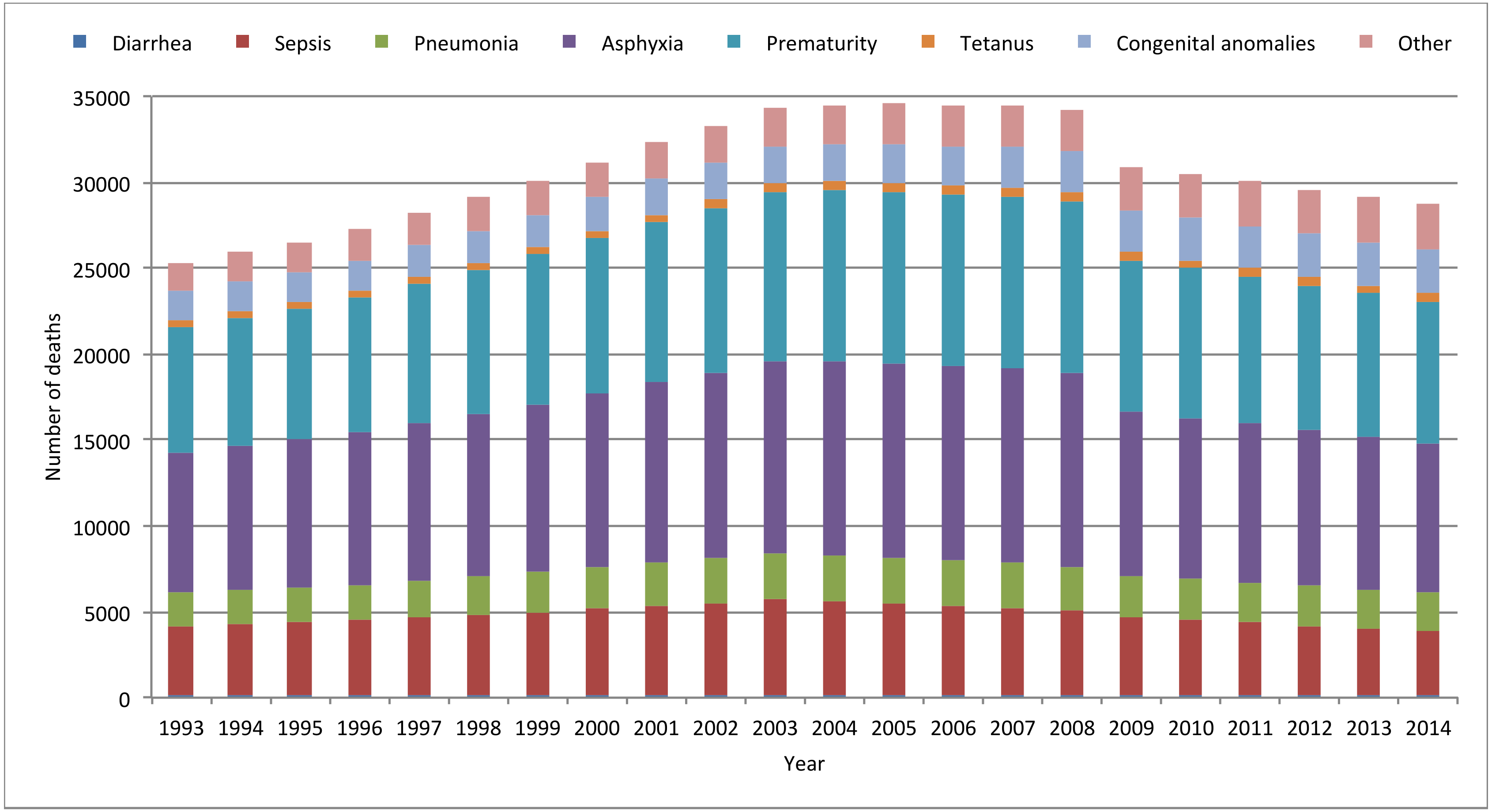

Figure 7: Trends in cause-specific under-five morality for the period 1993 to 2014.

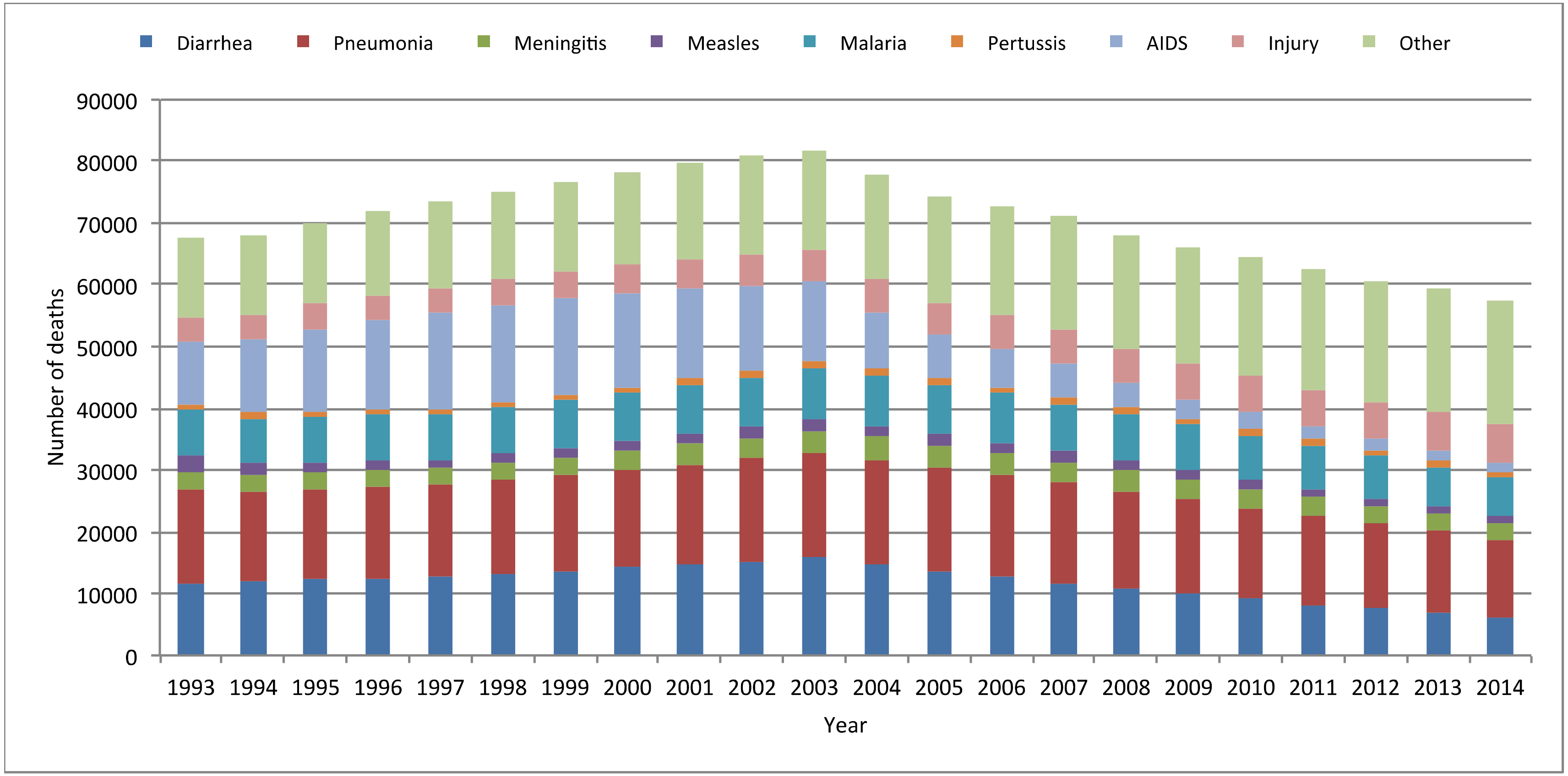

Figure 8: County-level change in intervention coverage from 2003 to 2014.

a. Coverage of family planning for women married/in union by county.

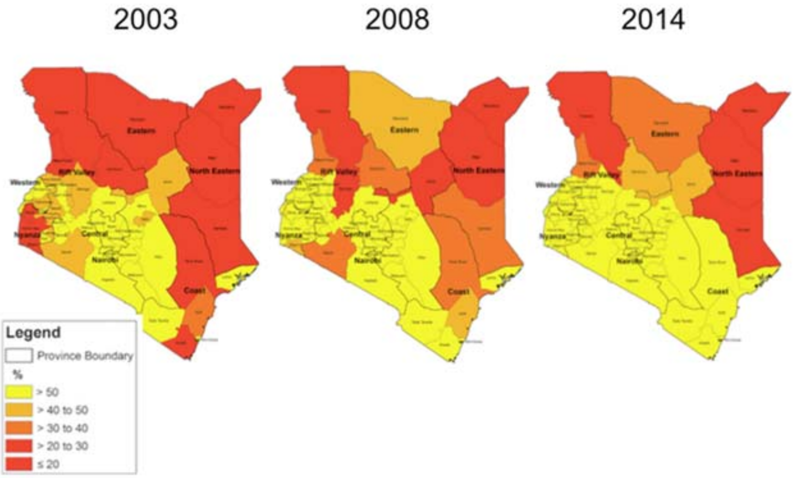

b. Coverage of skilled birth assistance (SBA) by county.

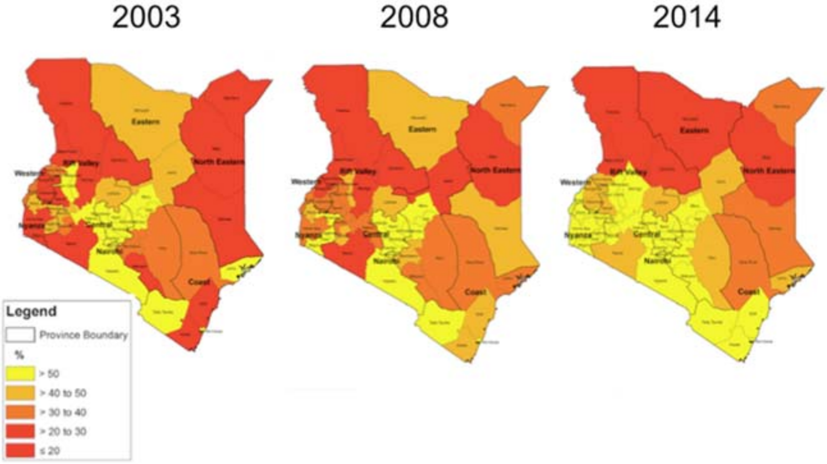

c. Coverage of full immunization by county.

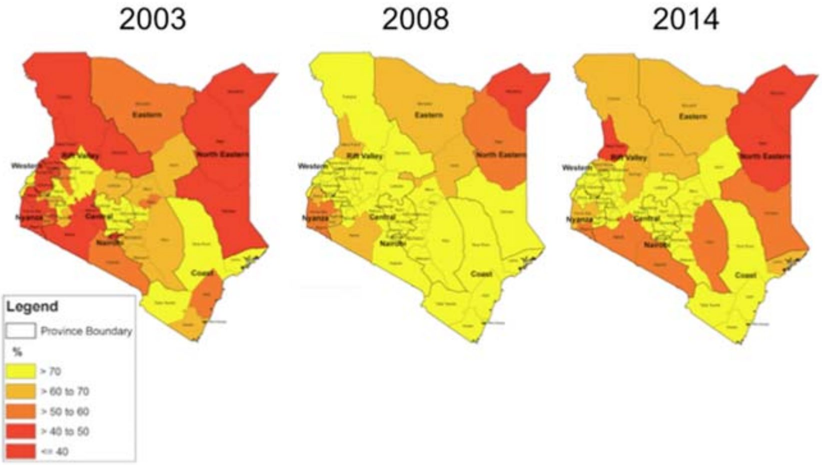

Figure 9: County-level coverage of composite coverage index (CCI) from 2003 to 2014.

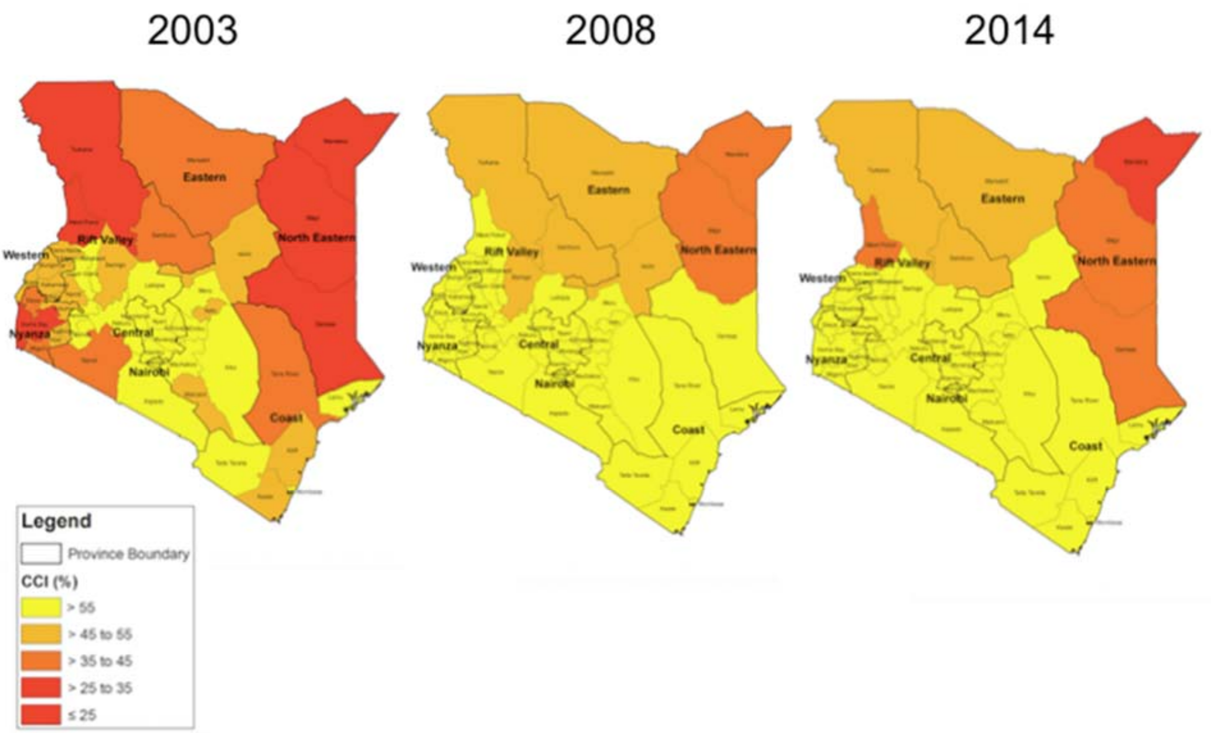

CCI: (i) demand for family planning satisfied, (ii) skilled birth attendance, (iii) antenatal care with a skilled provider, (iv) 3 doses of DTP vaccine, (v) measles vaccination, (vi) BCG vaccination, (vii) oral rehydration therapy and continued feeding for children with diarrhea, and (viii) care seeking for children with suspected pneumonia.

Figure 10: Number of maternal (a), neonatal (b), and post-neonatal (under-five) (c) deaths averted by specific intervention for the period 2003 to 2014.

a) Maternal deaths averted.

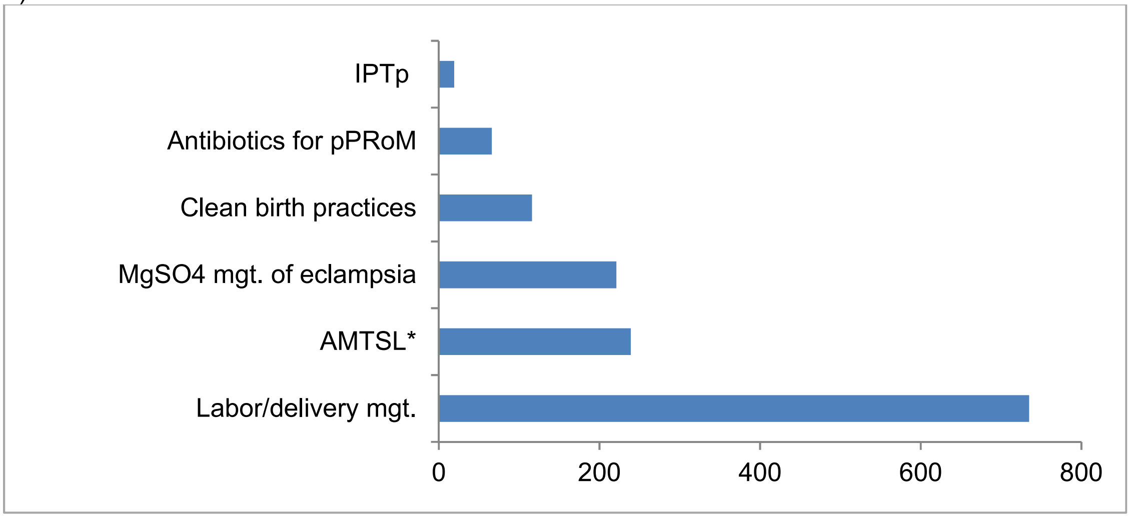

IPTp = intermittent preventive treatment for malaria in pregnancy; pPRoM = preterm premature rupture of membranes; MgSO4 = magnesium sulphate; AMTSL = active management of third stage of labour.

b) Neonatal deaths averted.

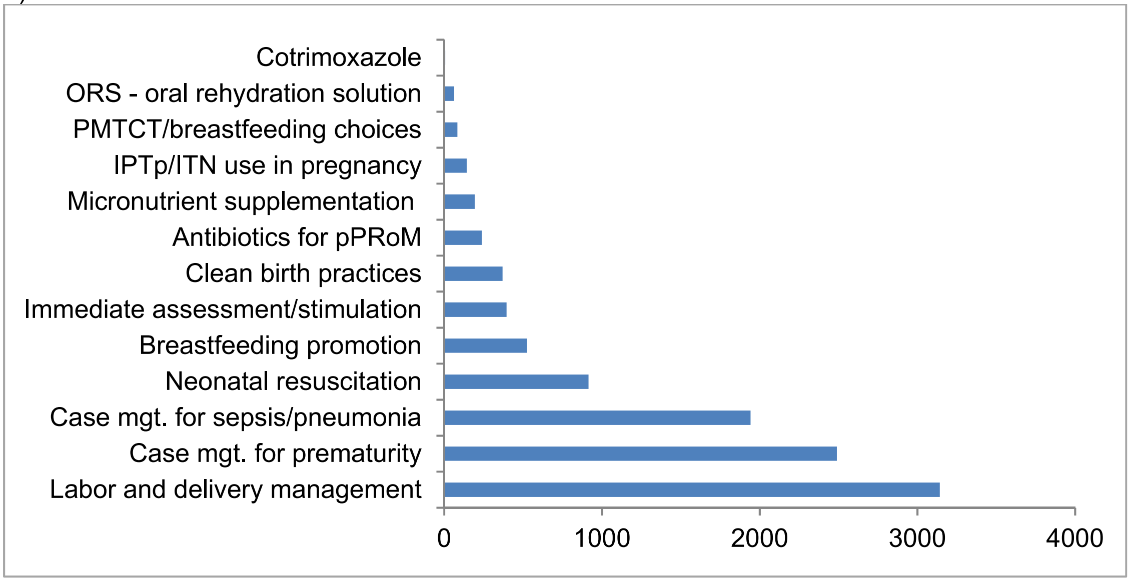

PMTCT = prevention of mother to child transmission of HIV; IPTp = intermittent preventive treatment for malaria in pregnancy; ITN = insecticide-treated net; pPRoM = preterm premature rupture of membranes.

c) Post-neonatal deaths averted.

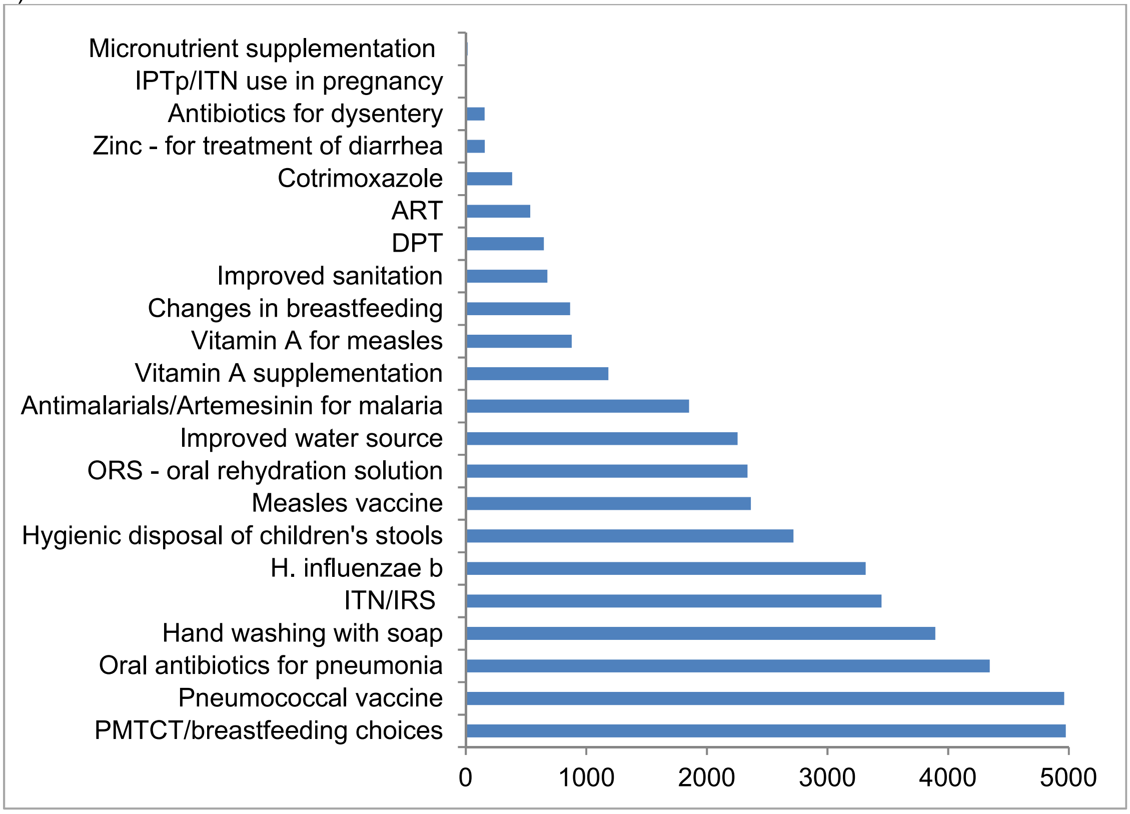

IPTp = intermittent preventive treatment for malaria in pregnancy; ITN = insecticide-treated net; ART = antiretroviral therapy; DPT = diphtheria, pertussis, tetanus vaccine; IRS = indoor residual spraying of insecticide; PMTCT = prevention of mother to child transmission of HIV.

Figure 11: Deaths prevented by scaling-up of community-driven interventions for each wealth quintile.

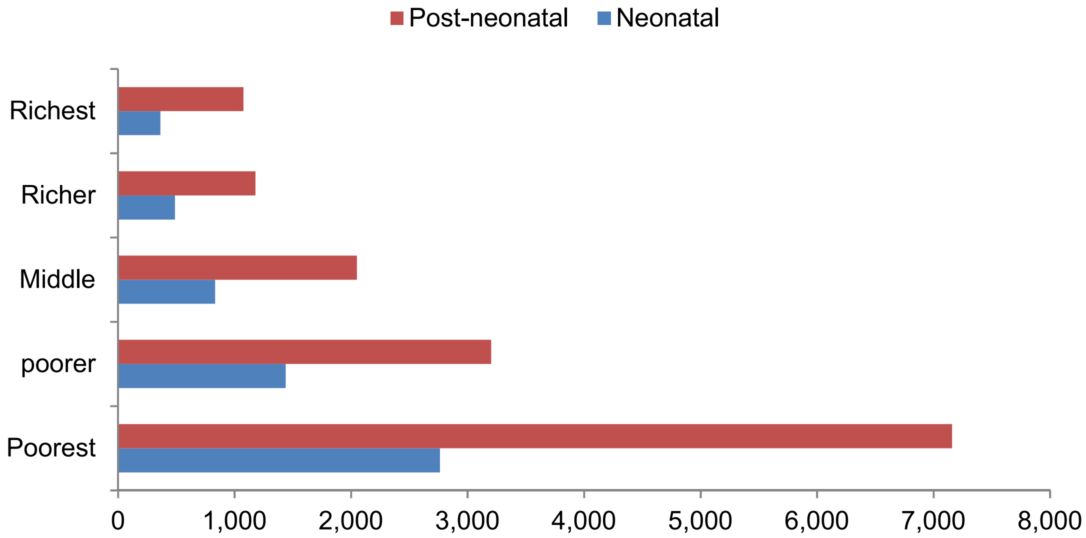

Supplement: Supplementary appendix [file mmc1.pdf]
